# Supplementary material for: Joint connectivity matrix independent component analysis: Auto‐linking of structural and functional connectivities
Source: Hum Brain Mapp. 2022 Nov 24;44(4):1533–47. doi: 10.1002/hbm.26155 (PMC9921228; doi:10.1002/hbm.26155)

**Supplemental**

1. Complete framework


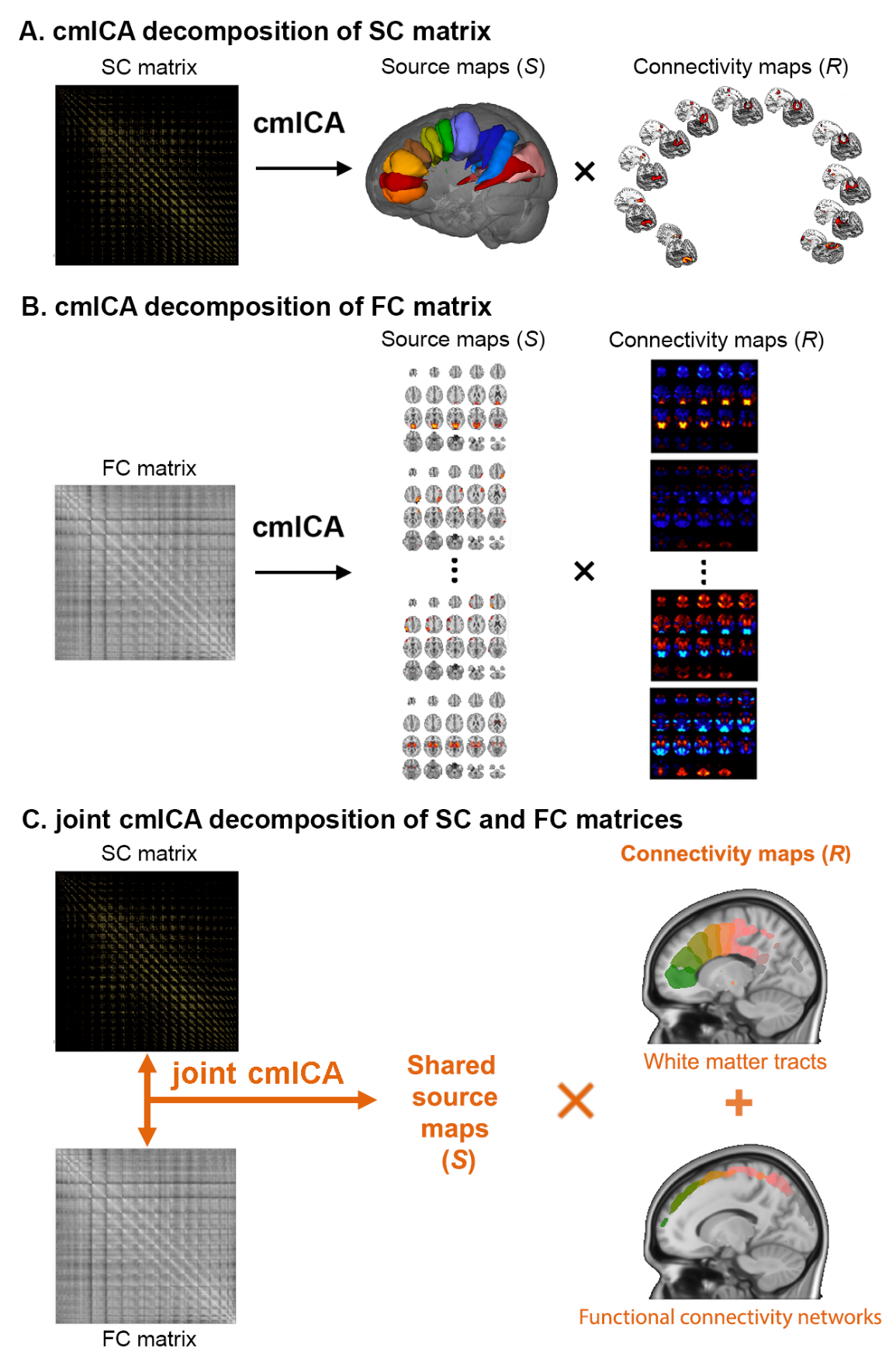


1. 60 source maps’ contribution from FC matrix and SC matrix


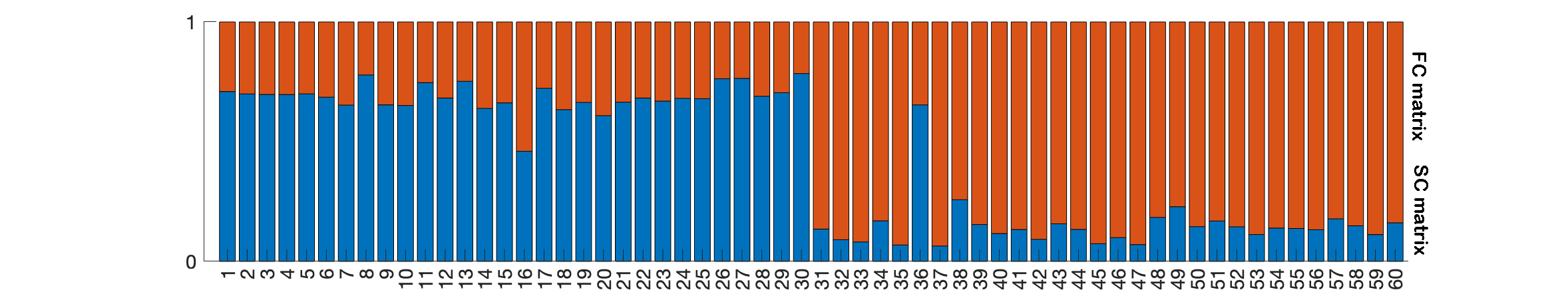


1. Joint cmICA *S* parcellations from FC matrix (‘red’) and SC matrix (‘blue’), all 60 components.


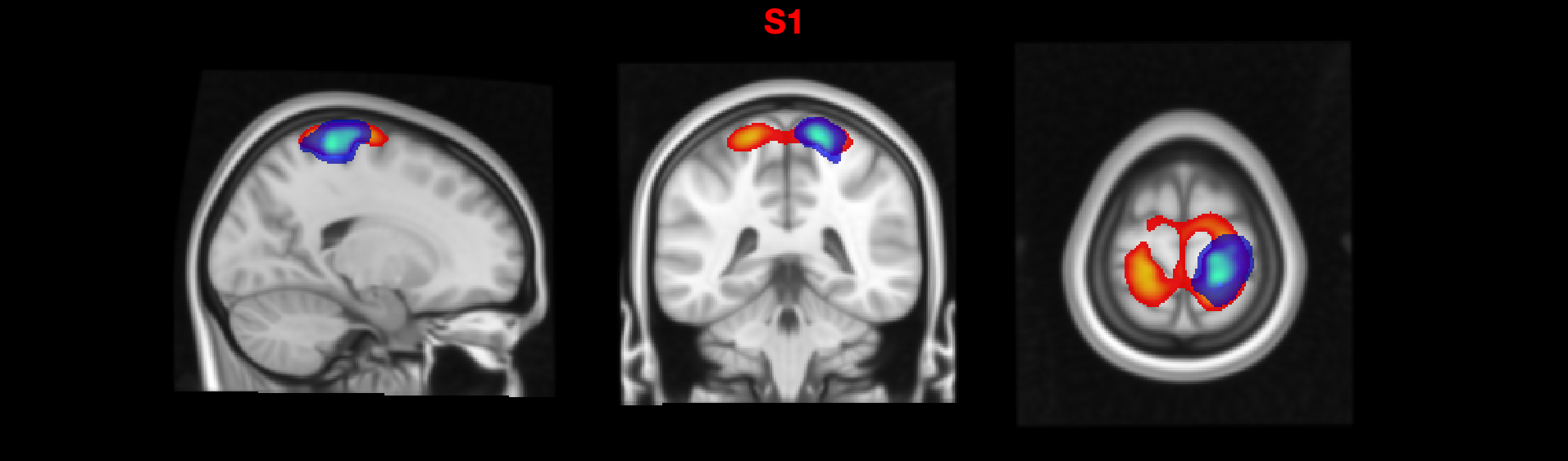

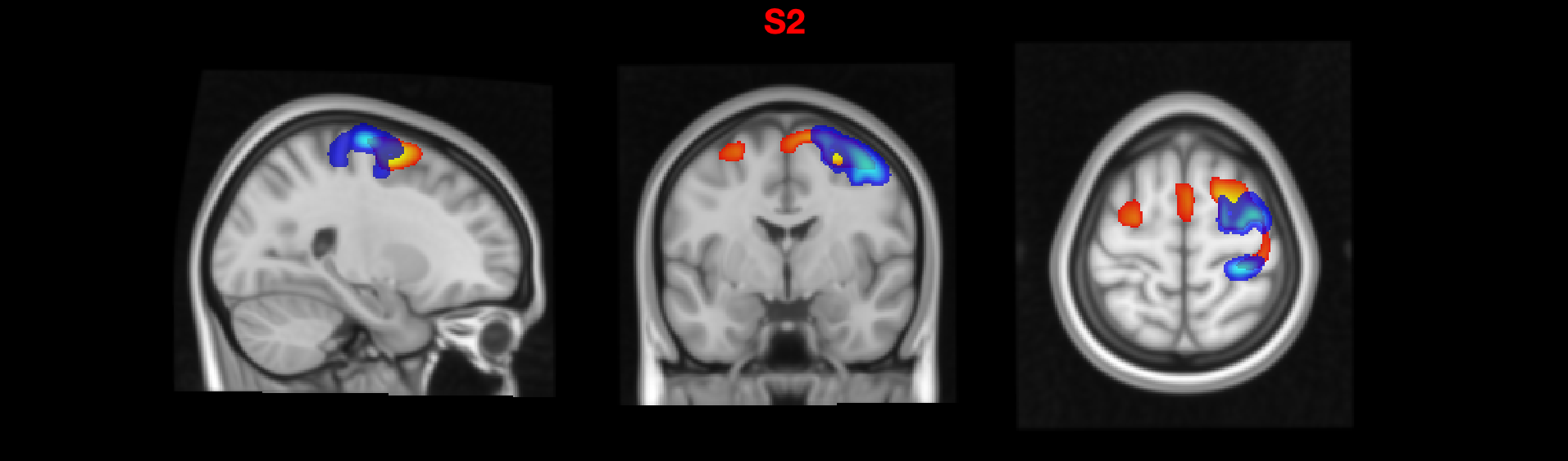

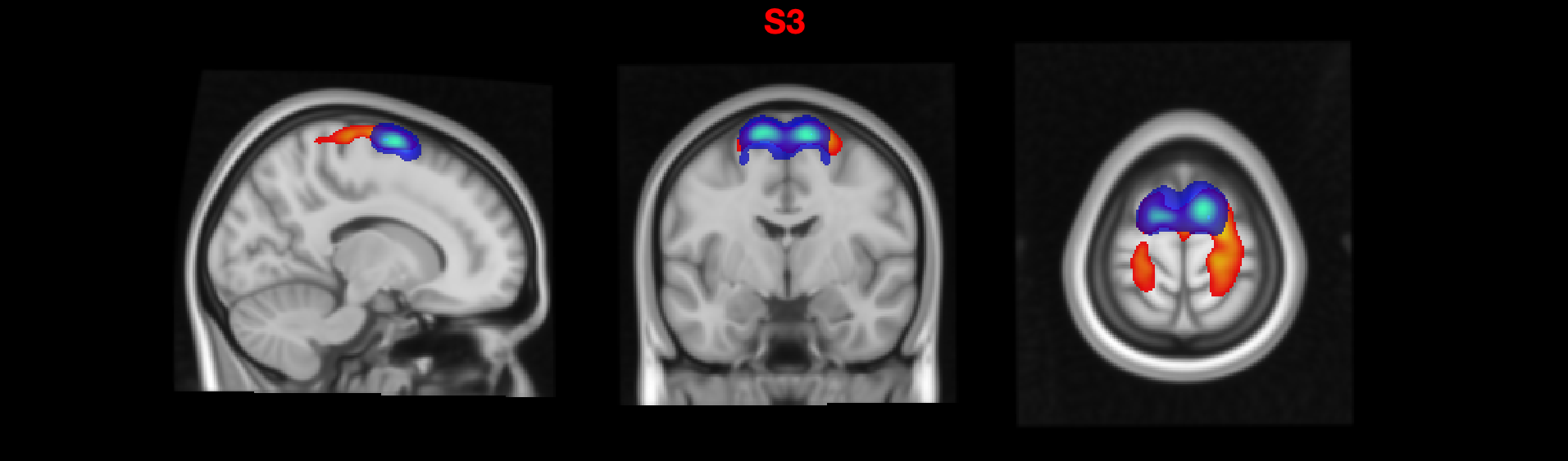

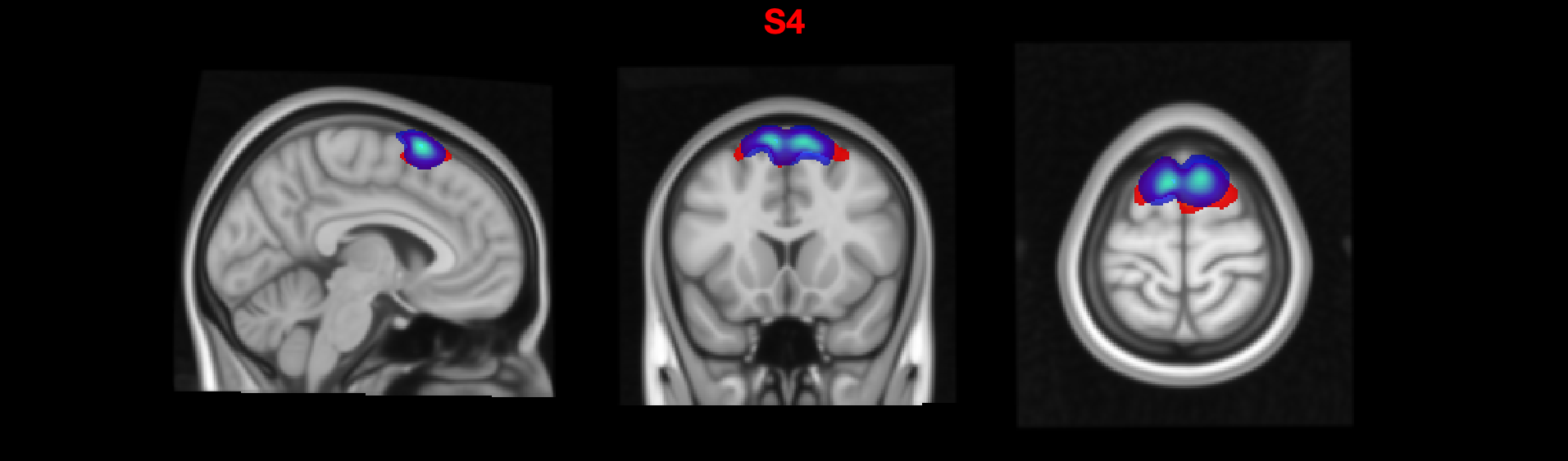

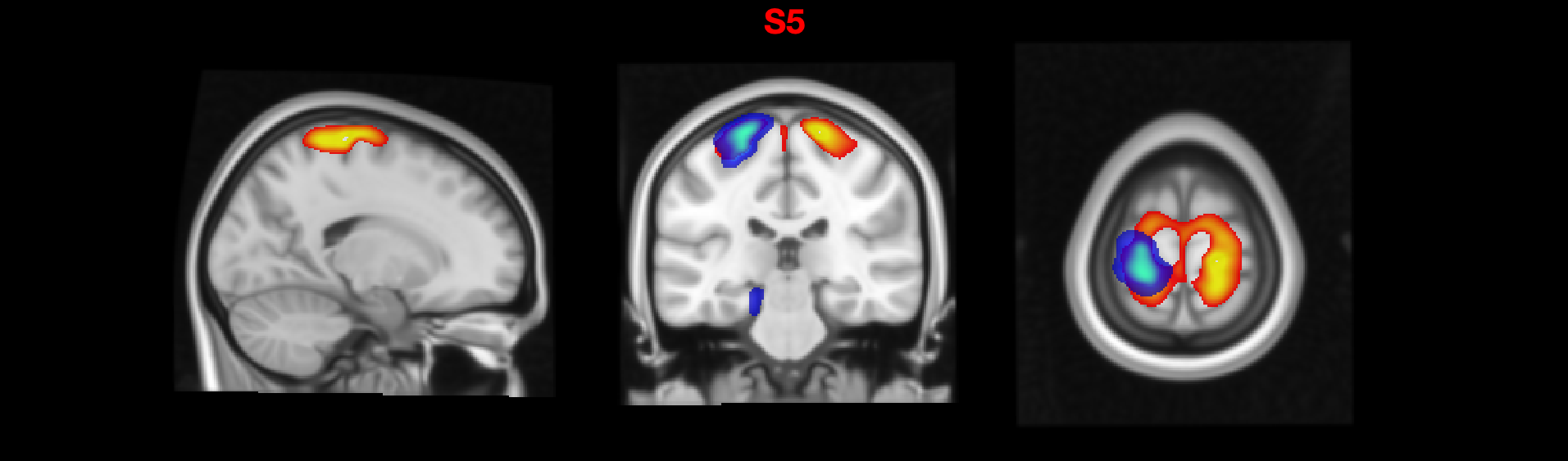

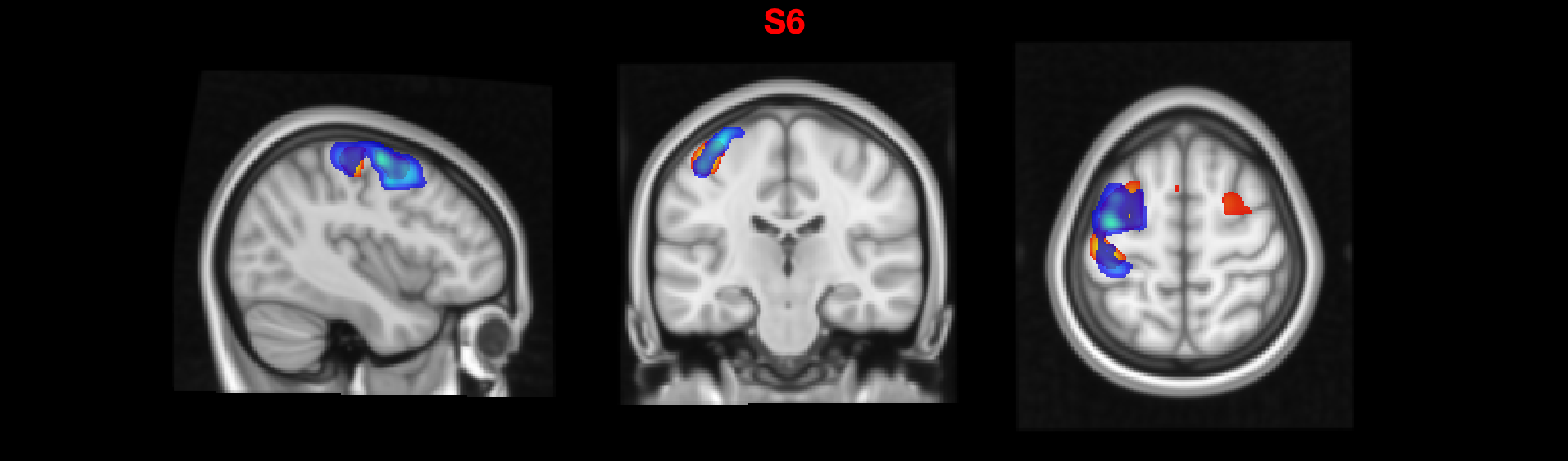

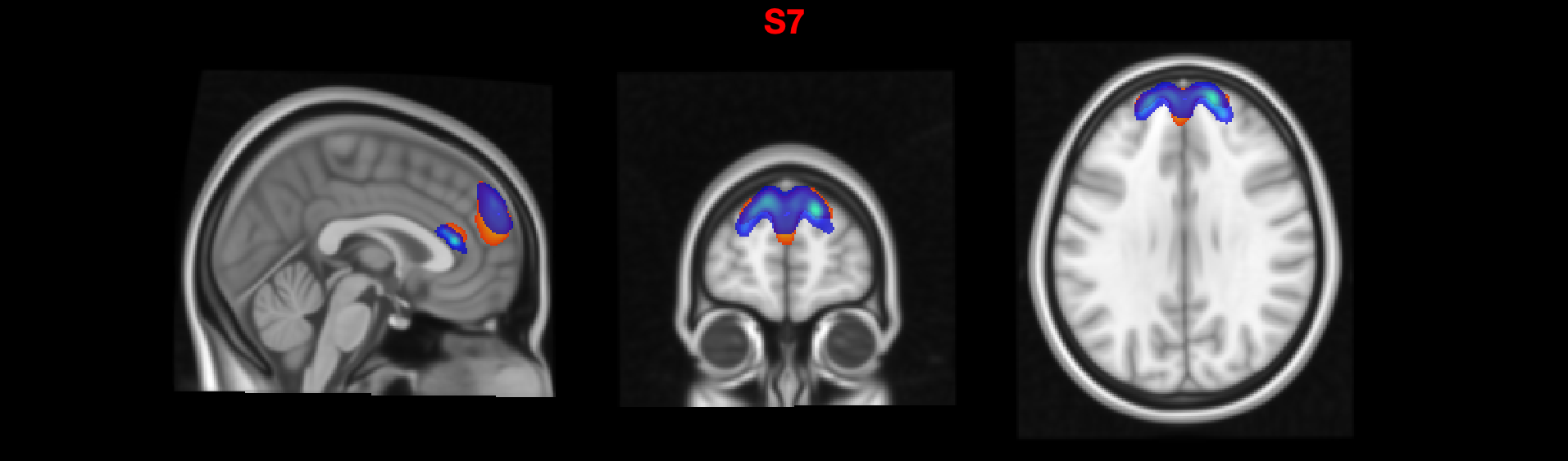

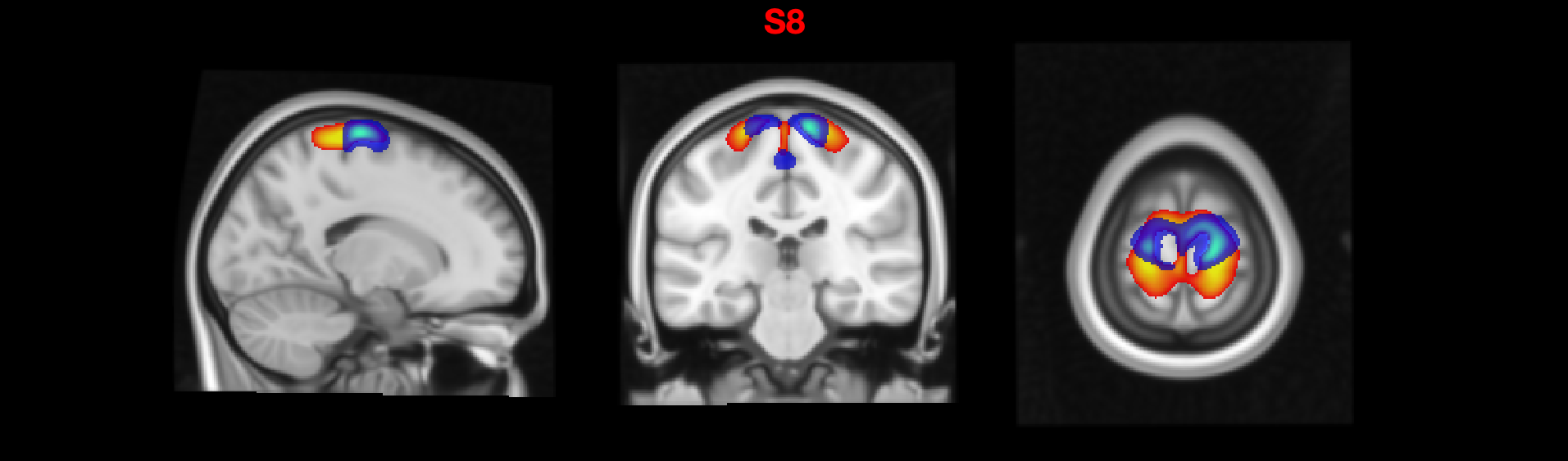

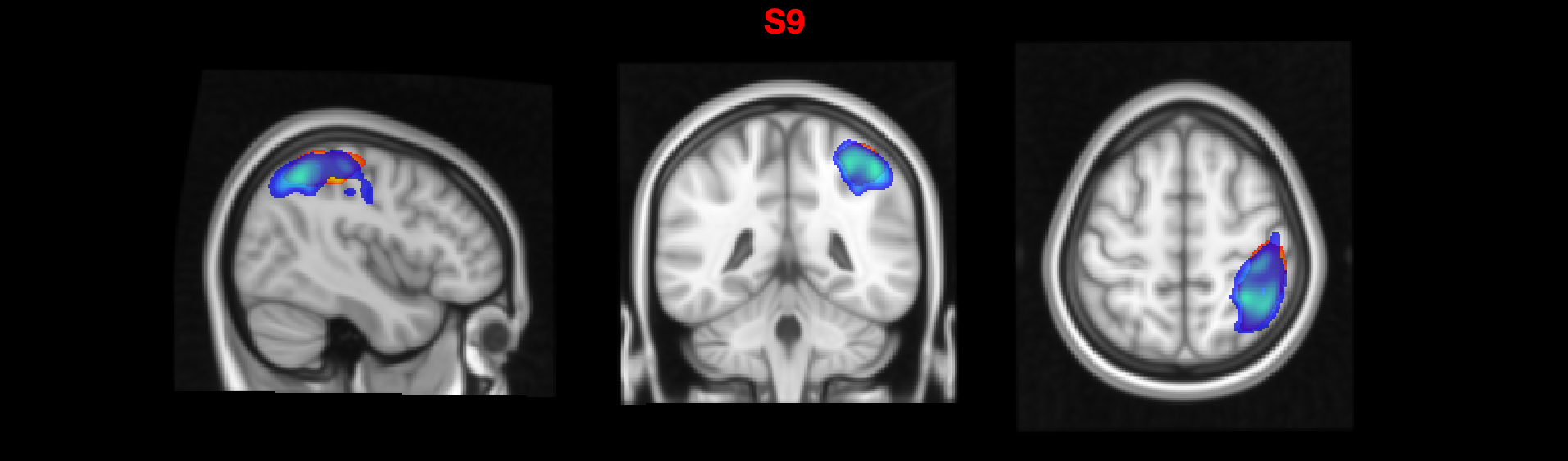

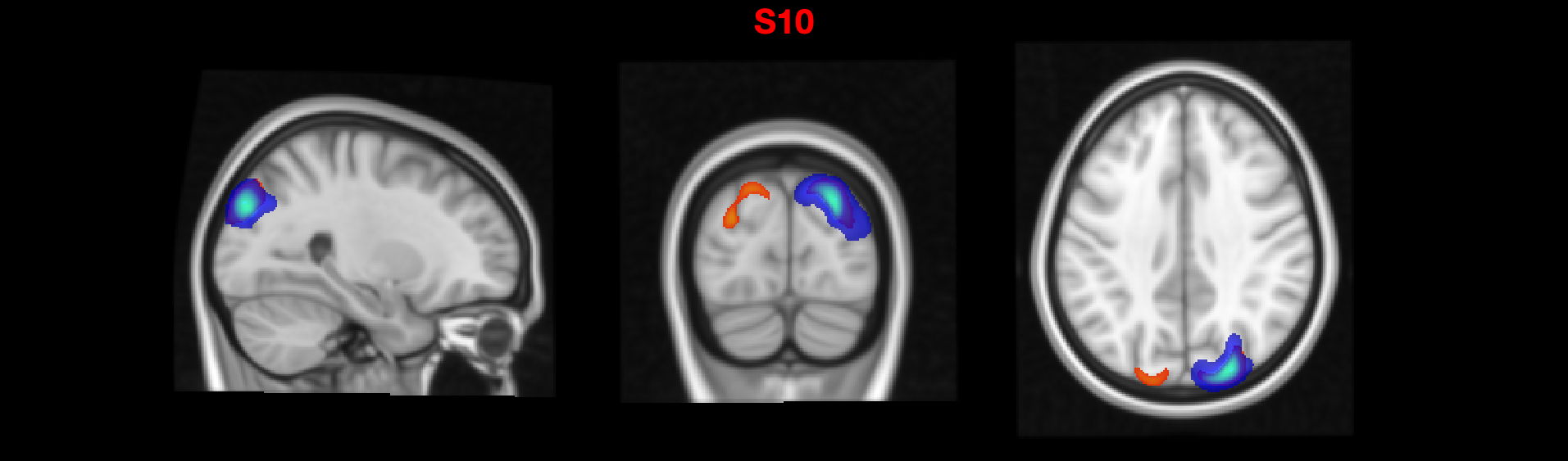

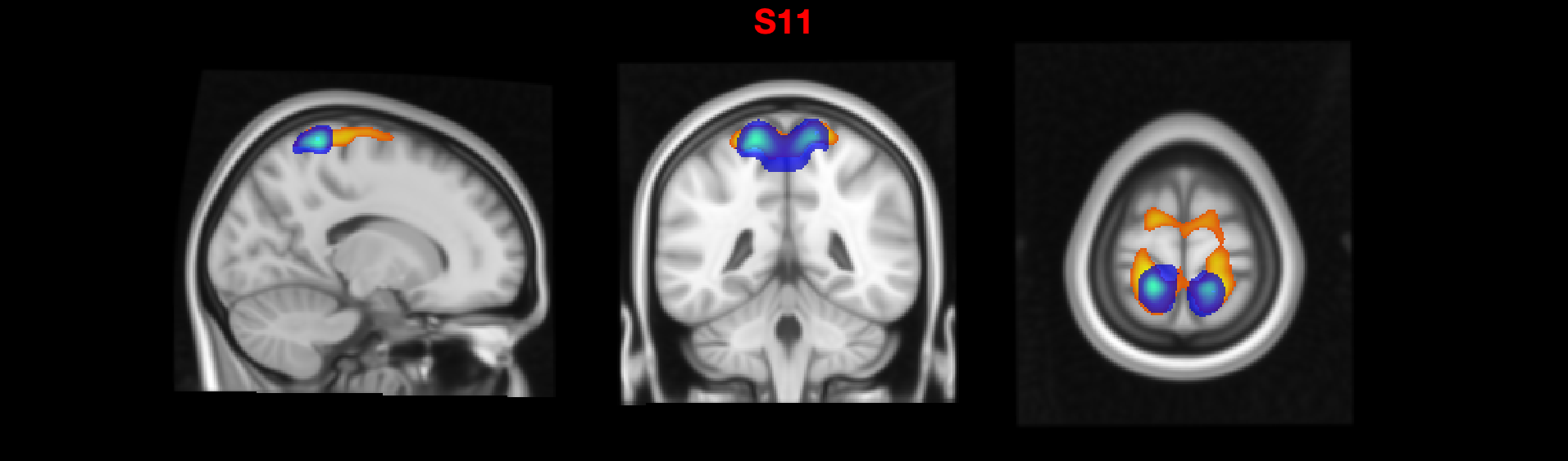

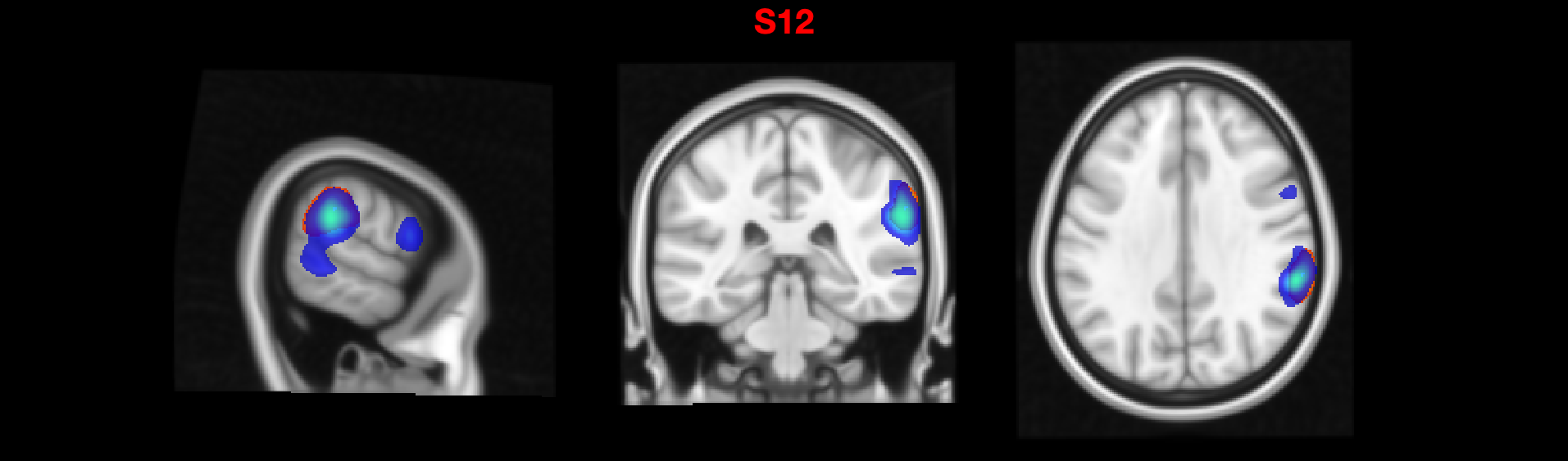

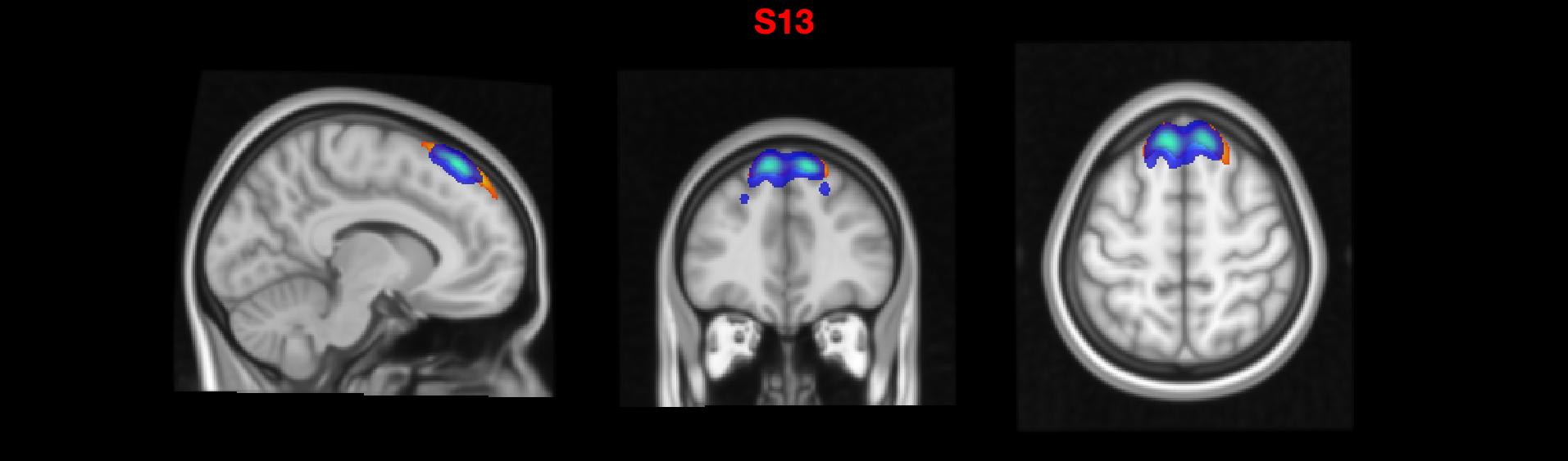

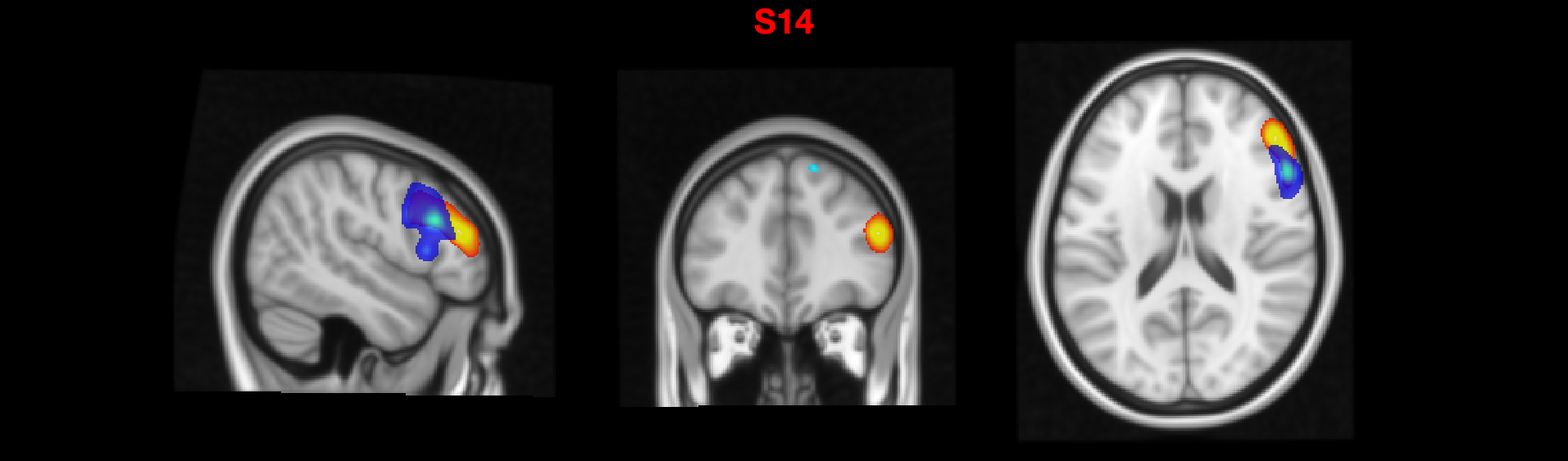

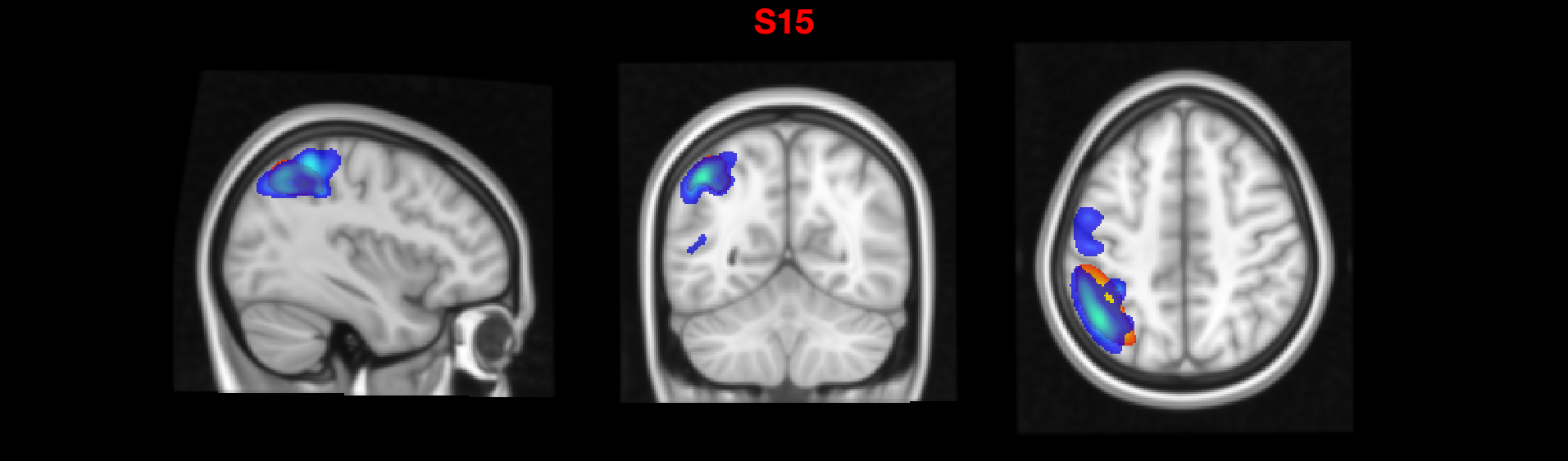

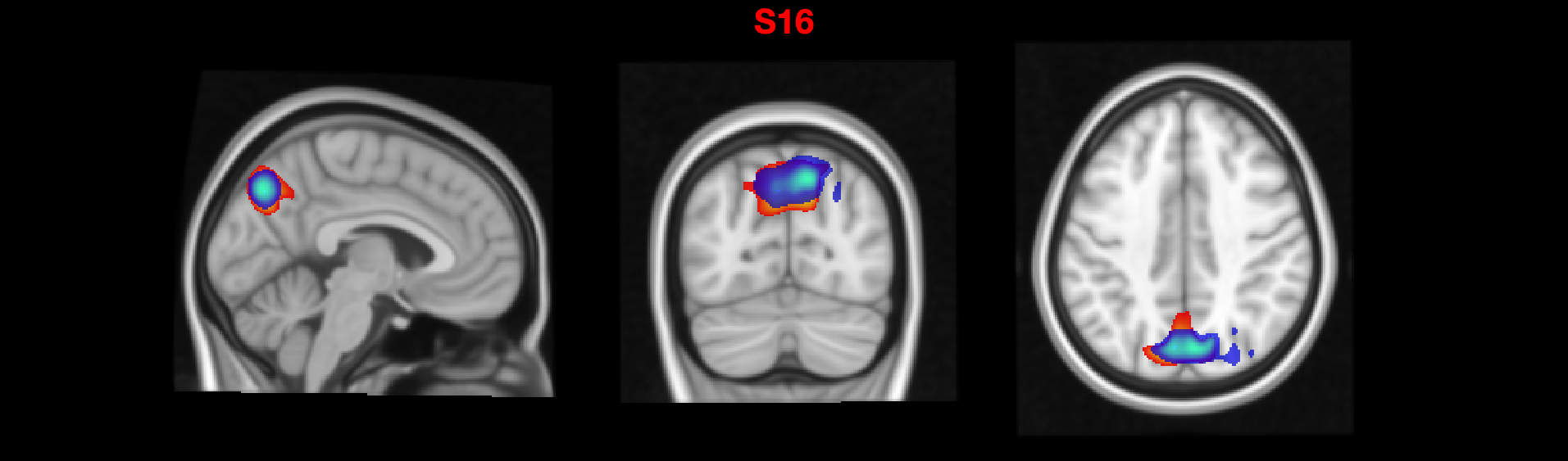

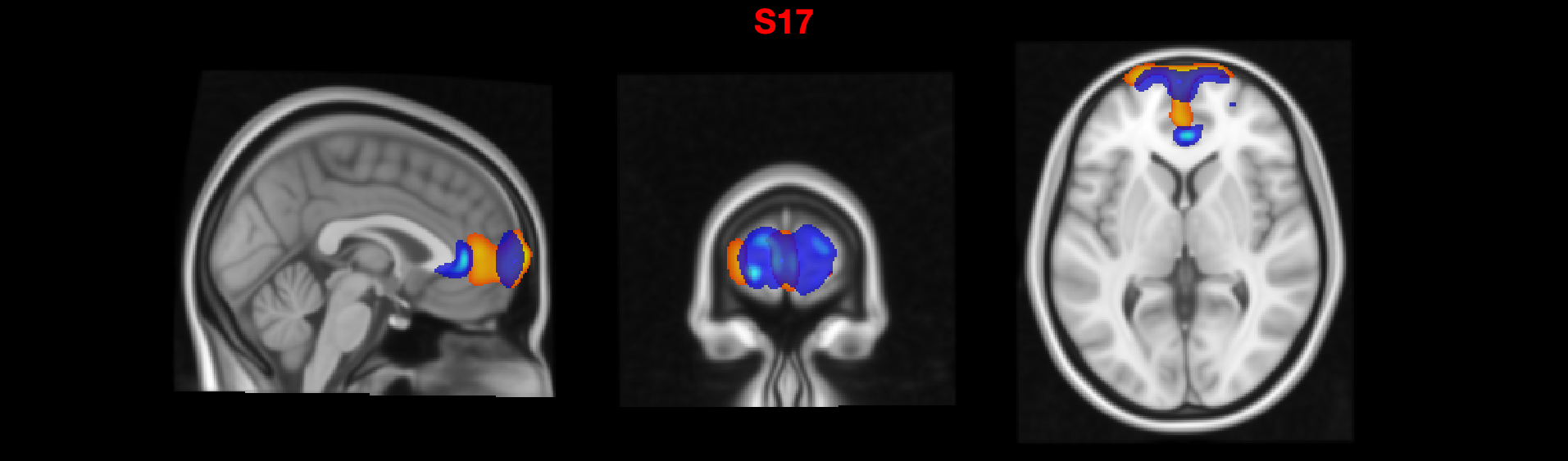

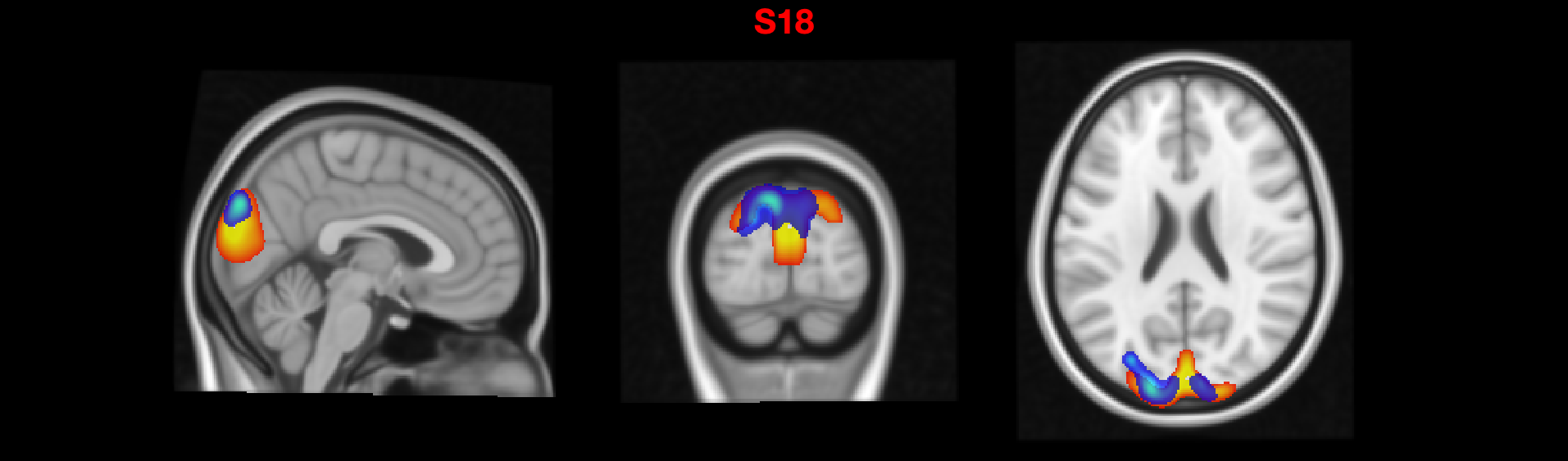

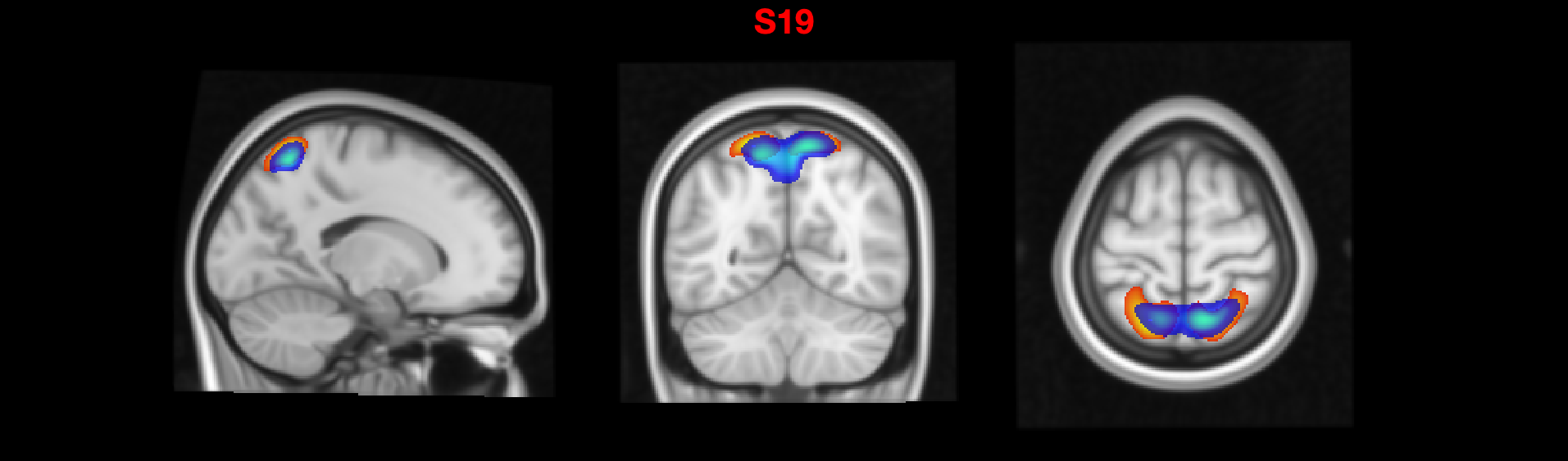

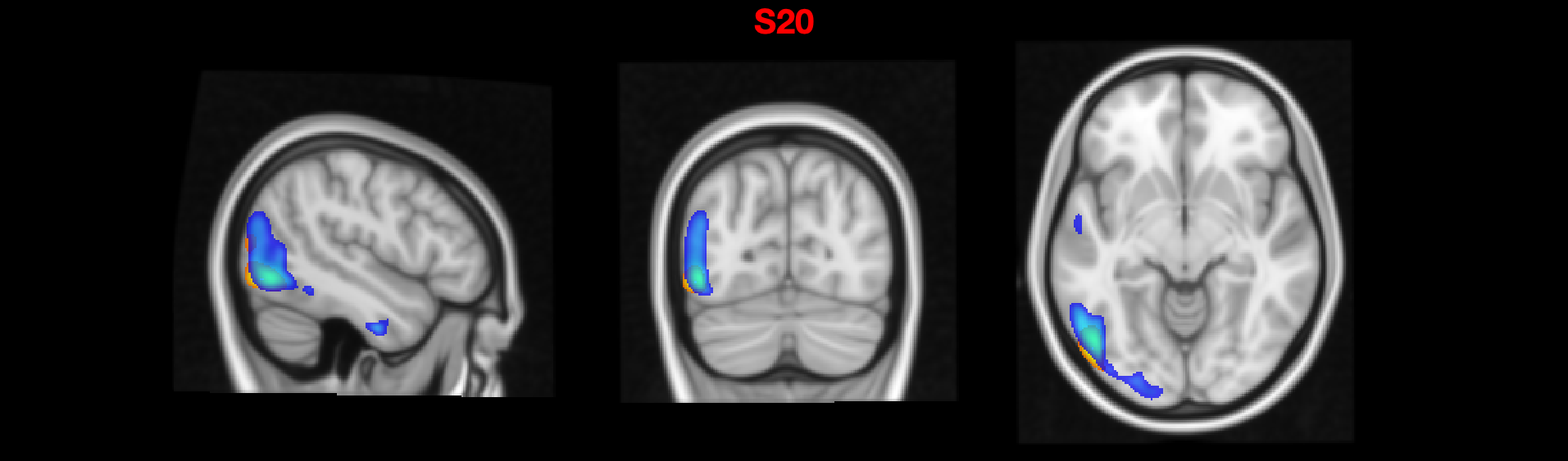

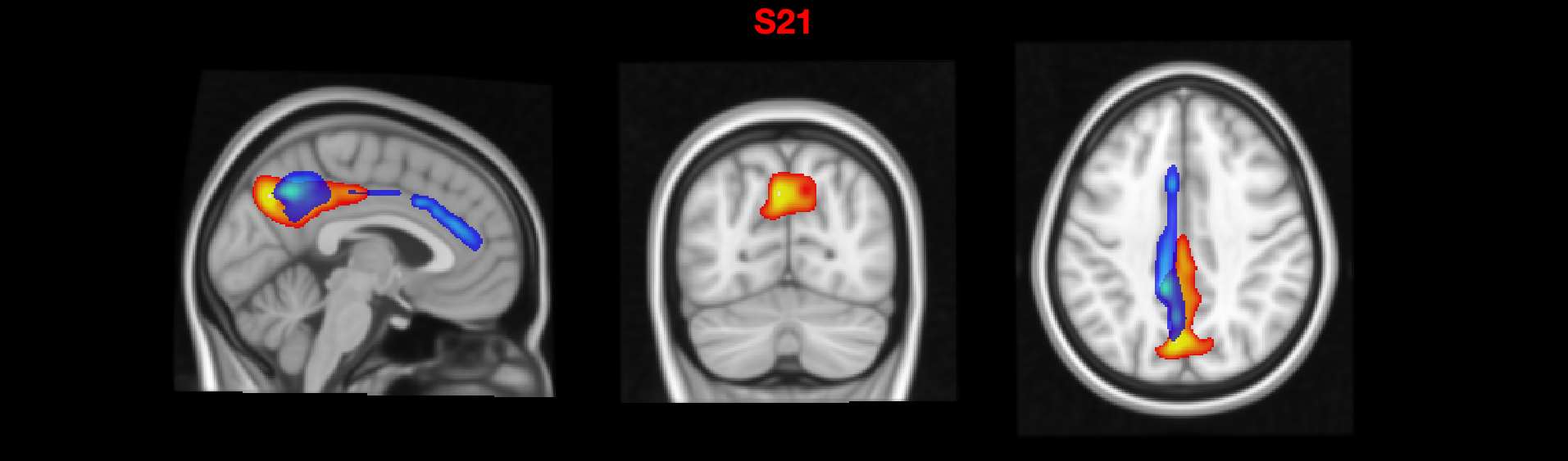

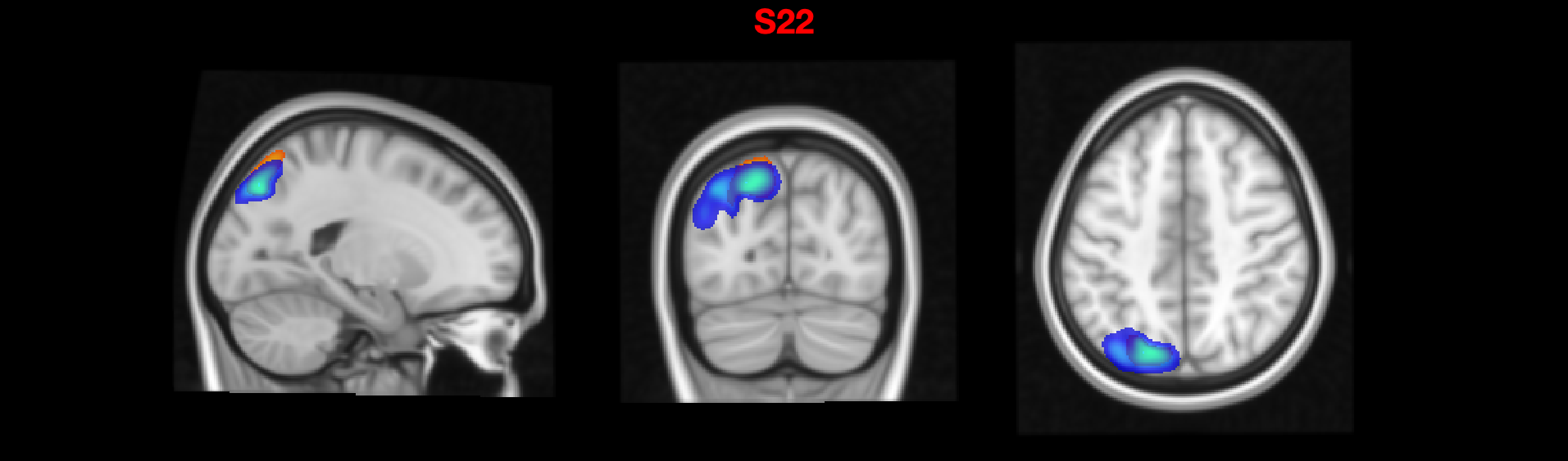

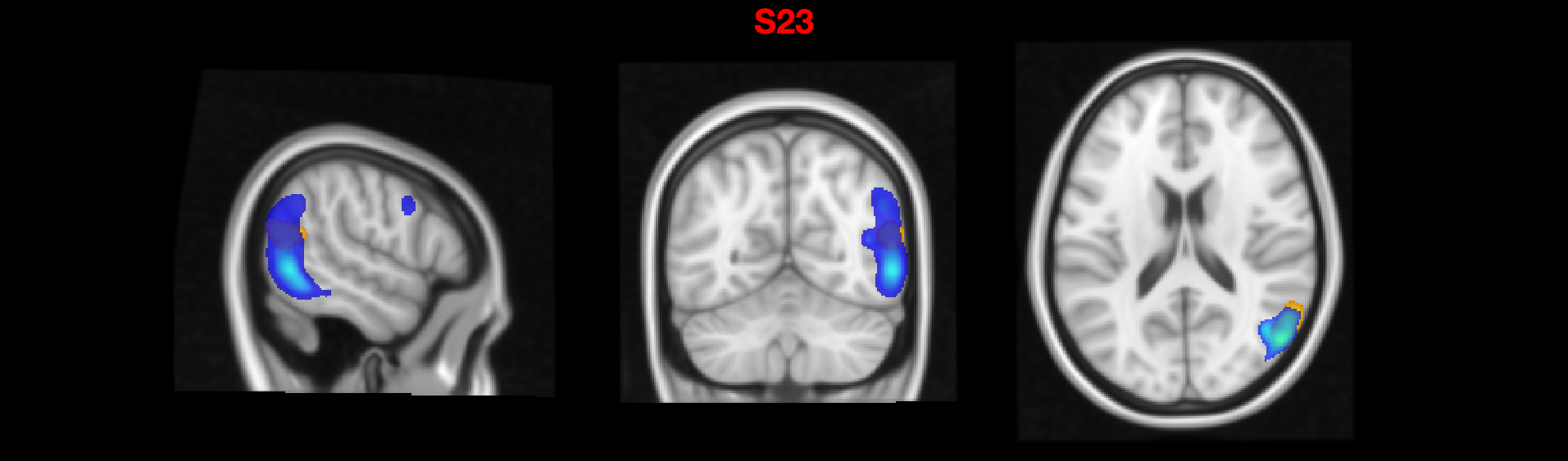

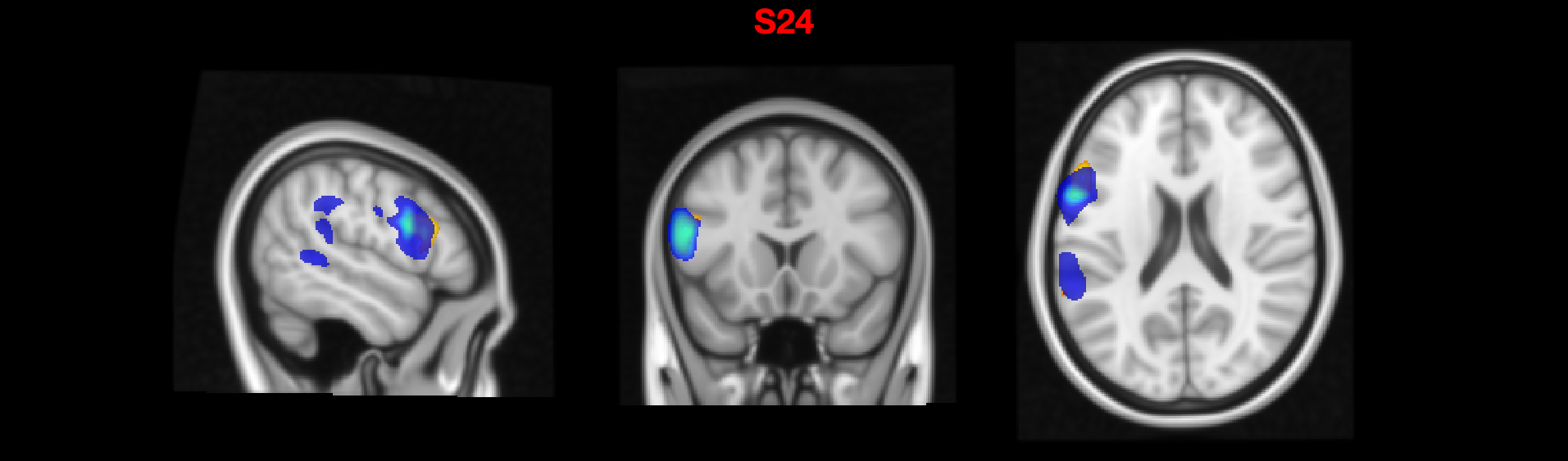

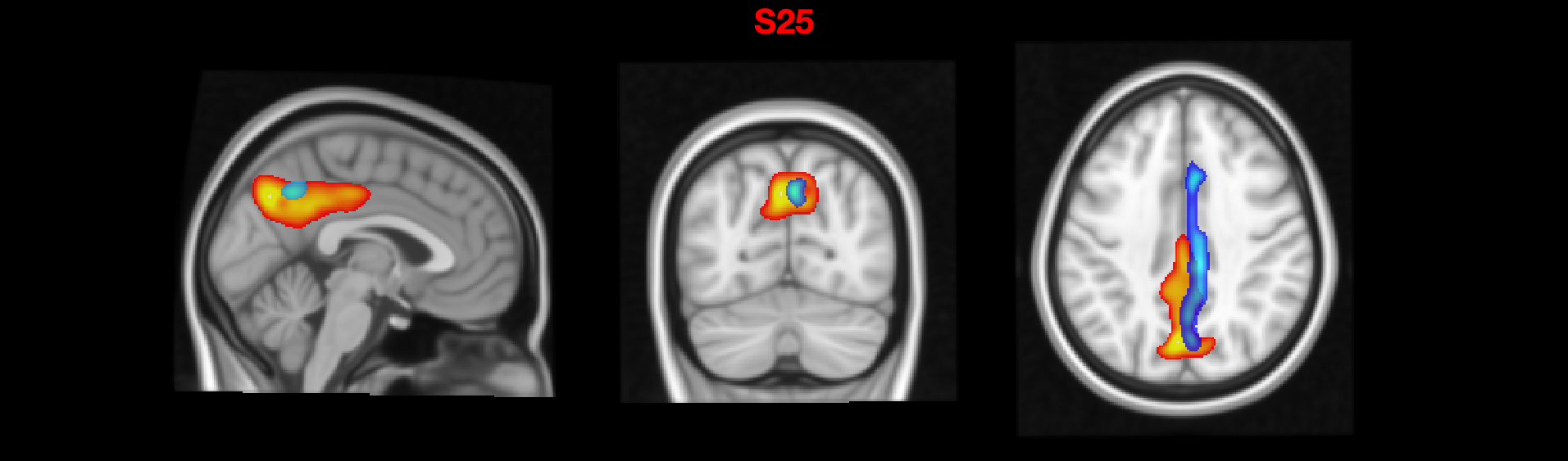

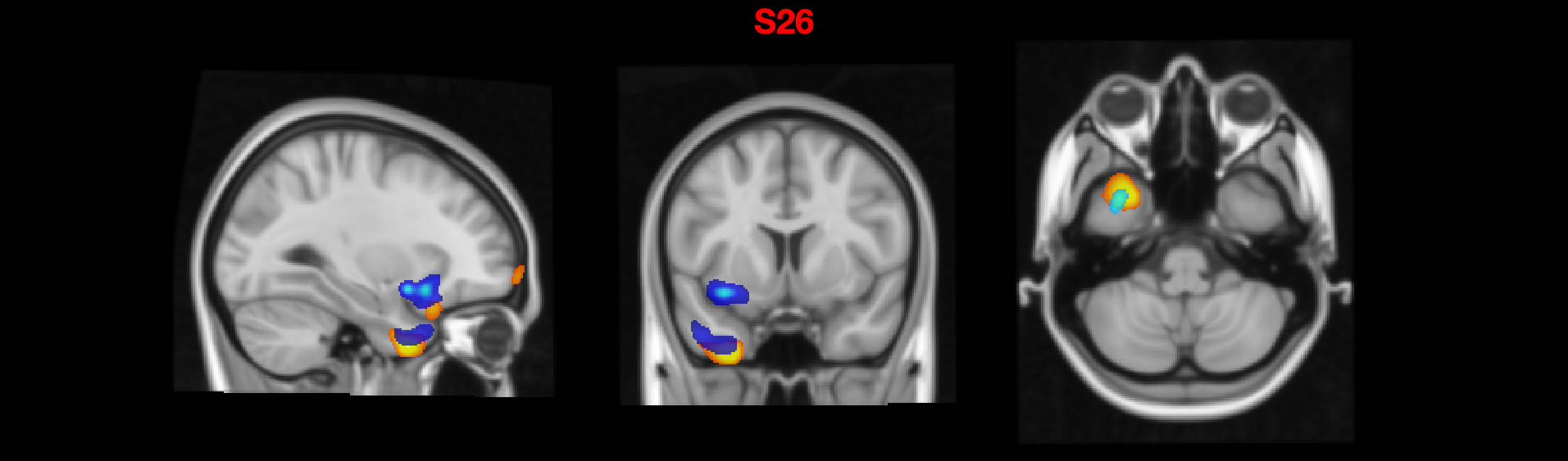

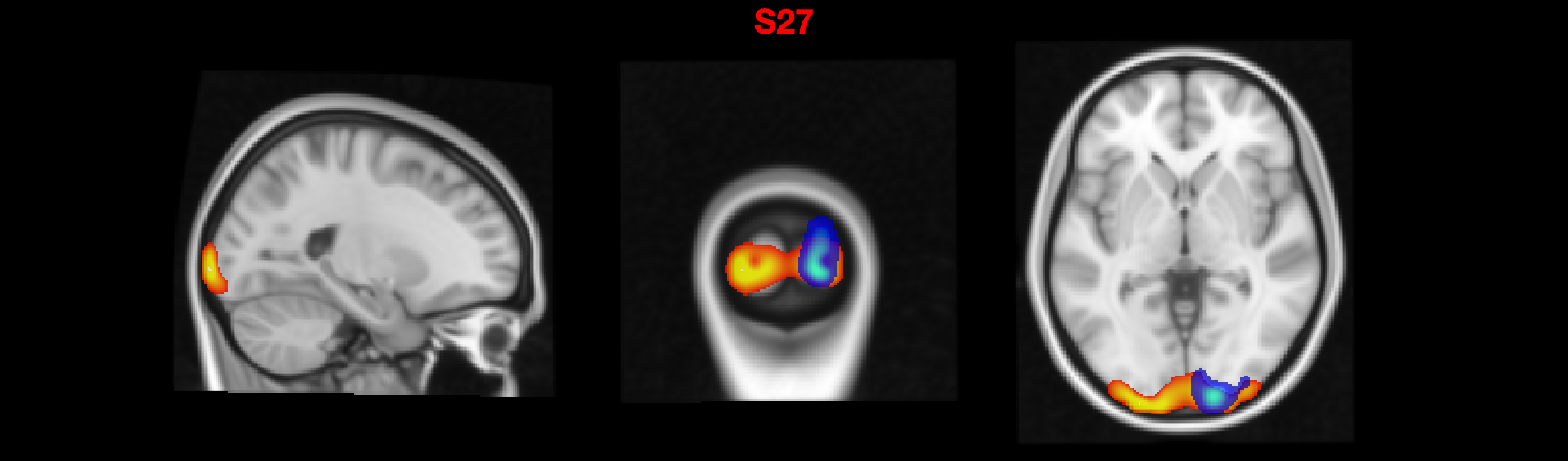

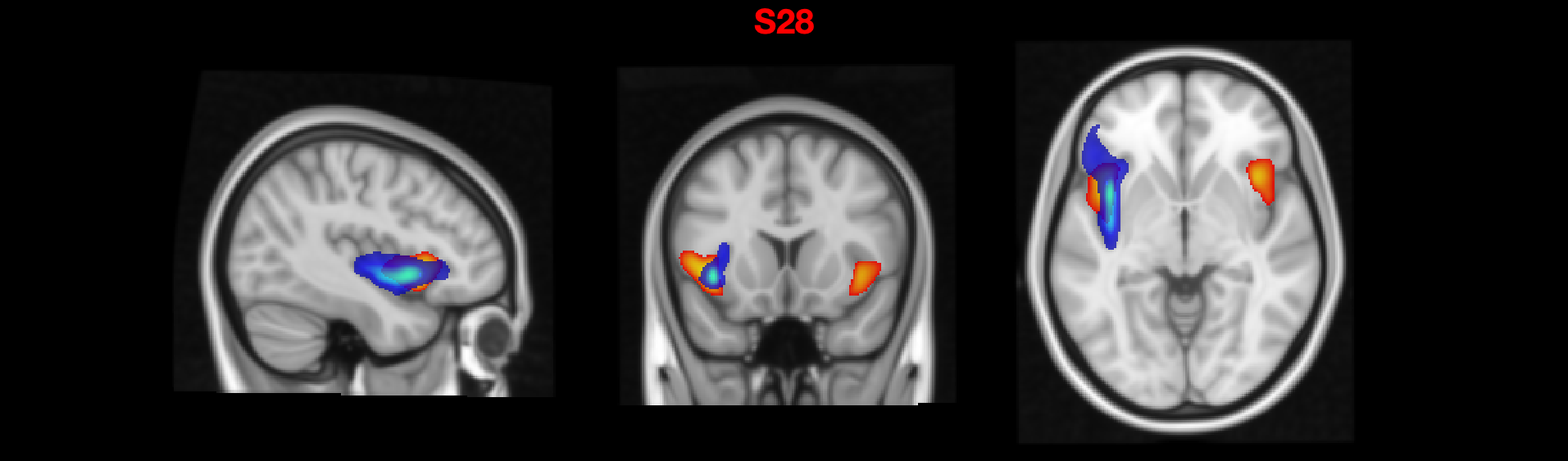

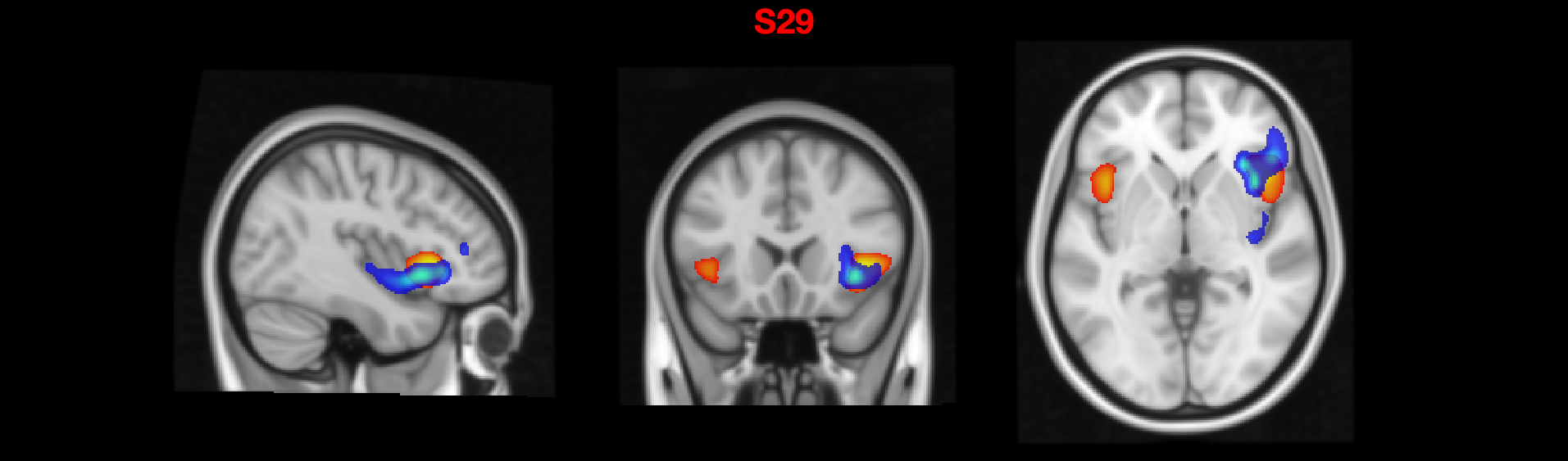

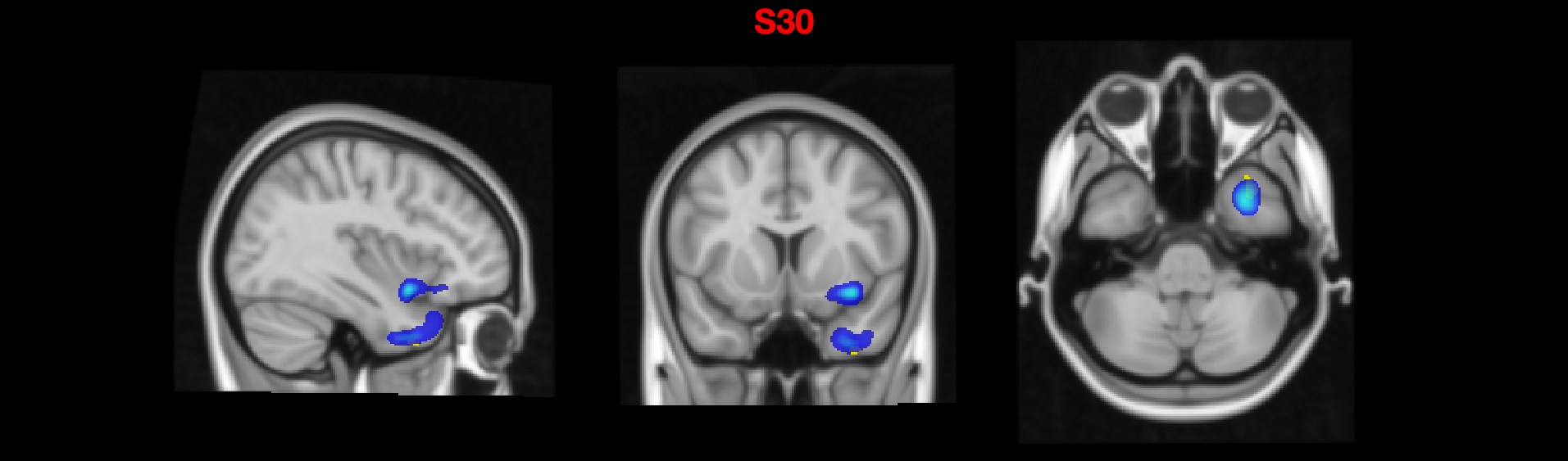


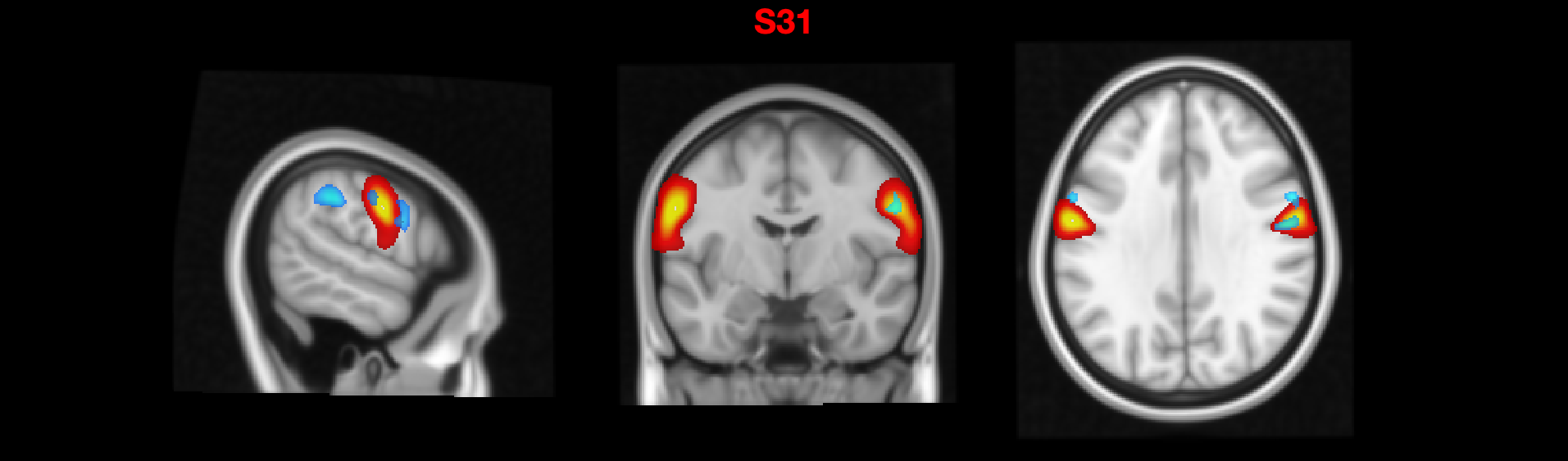

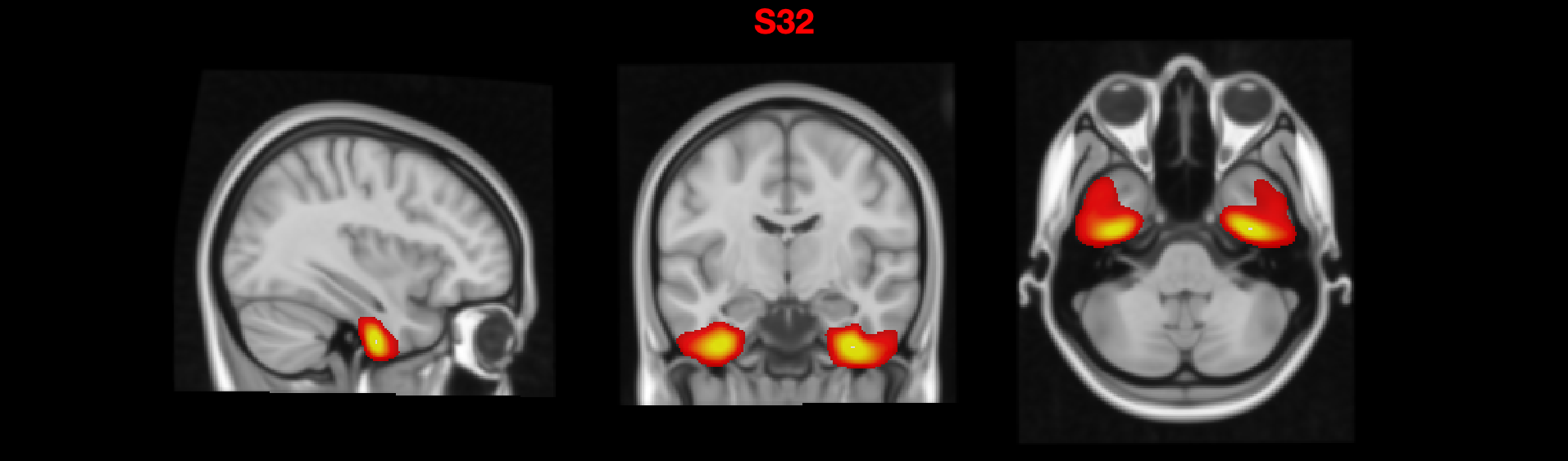

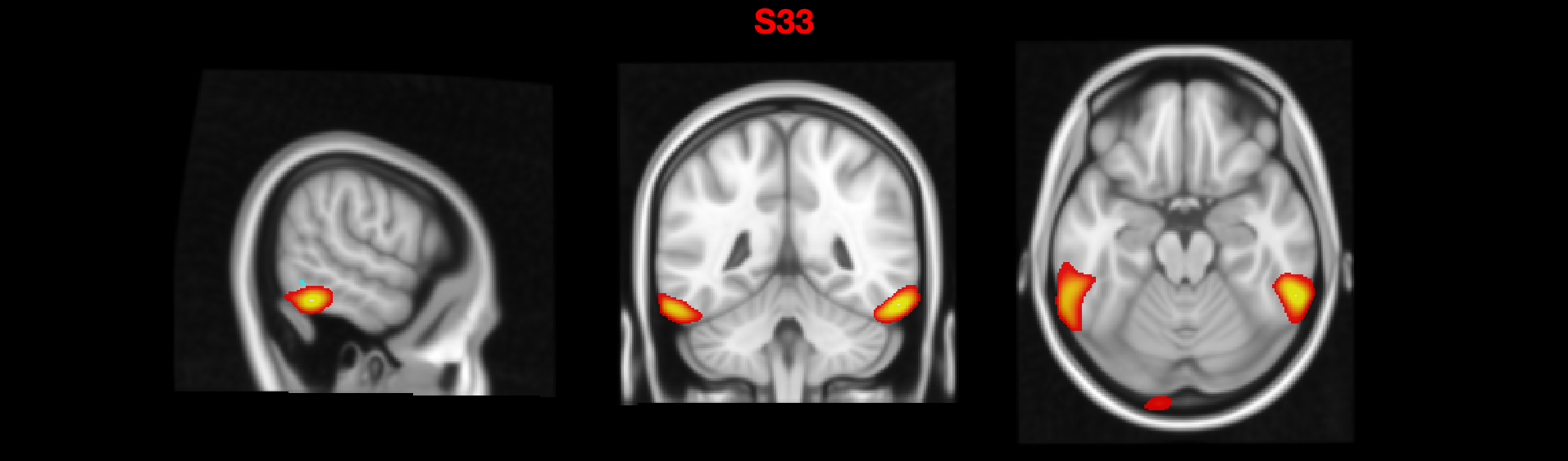

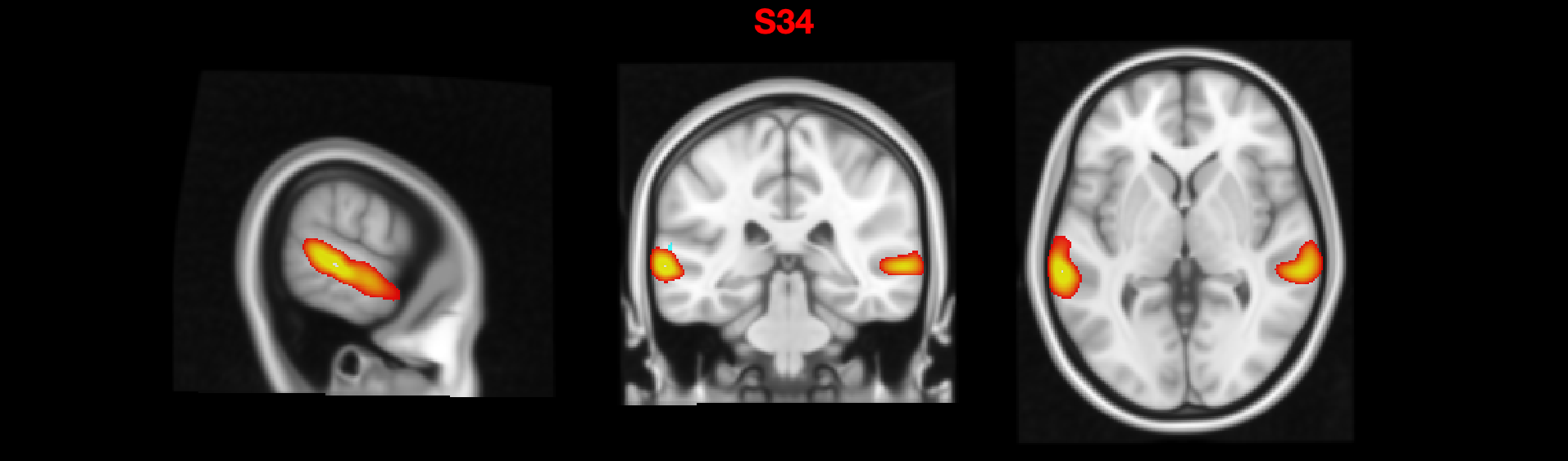

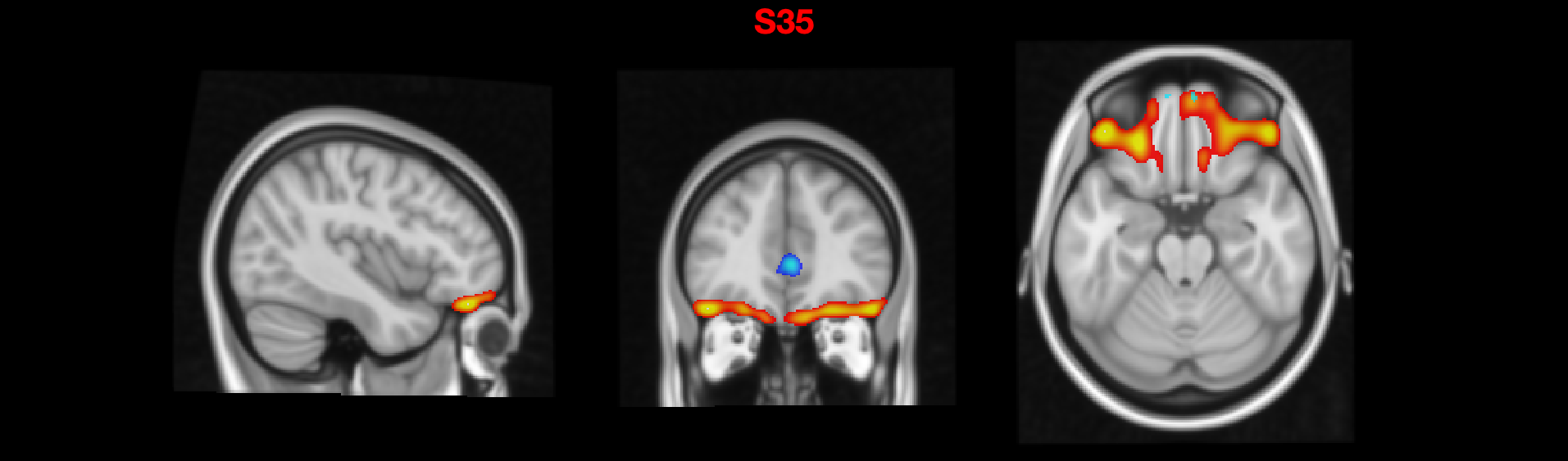

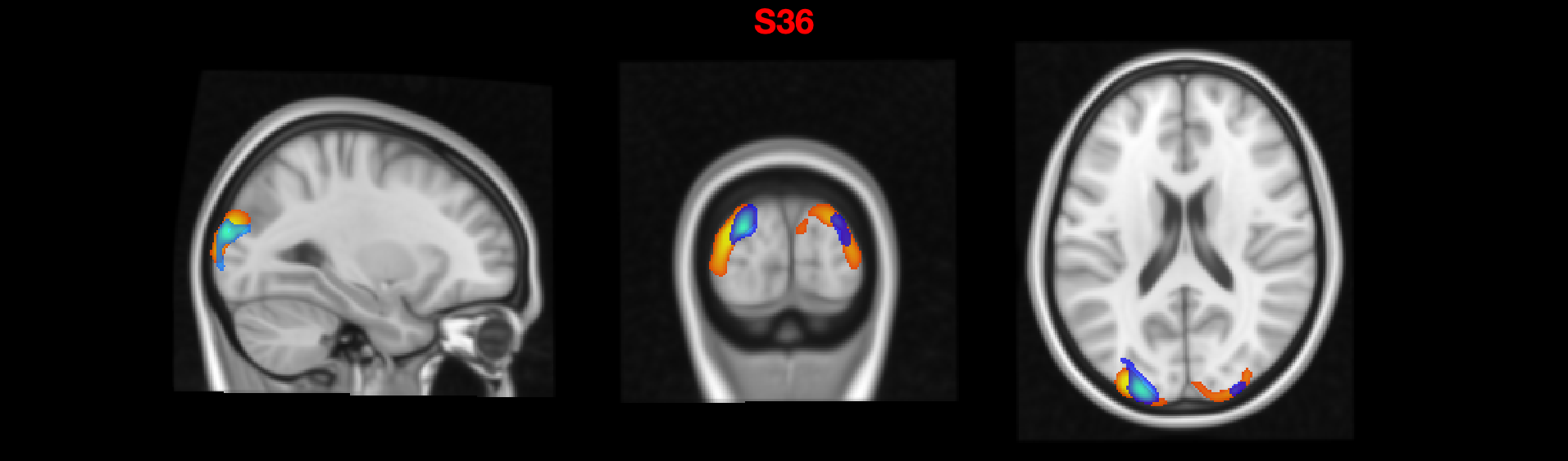

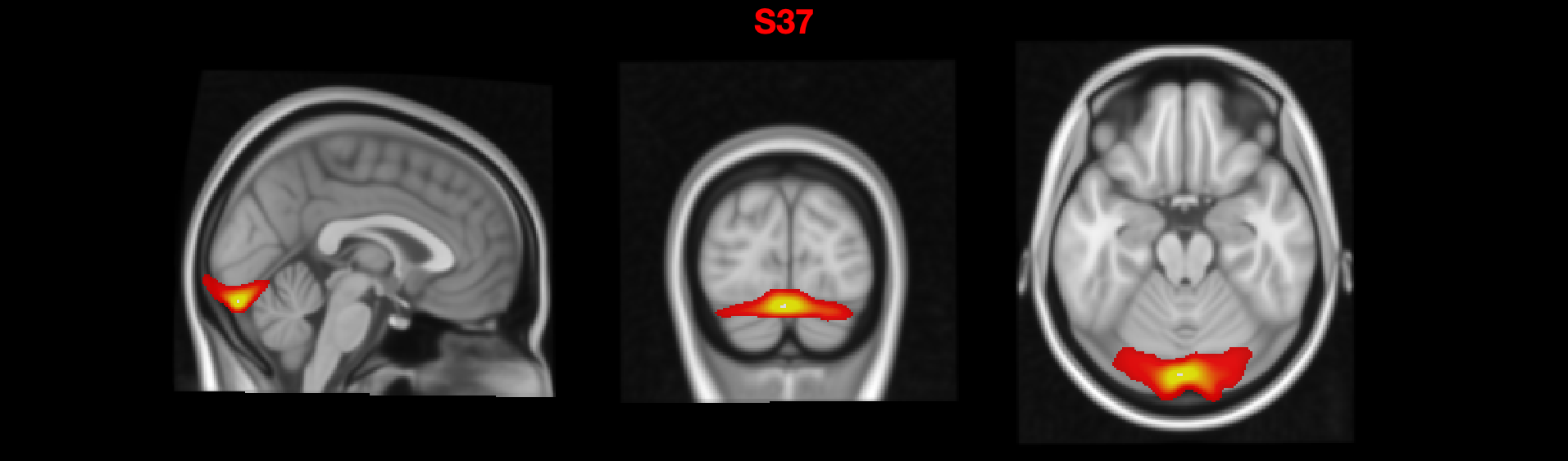

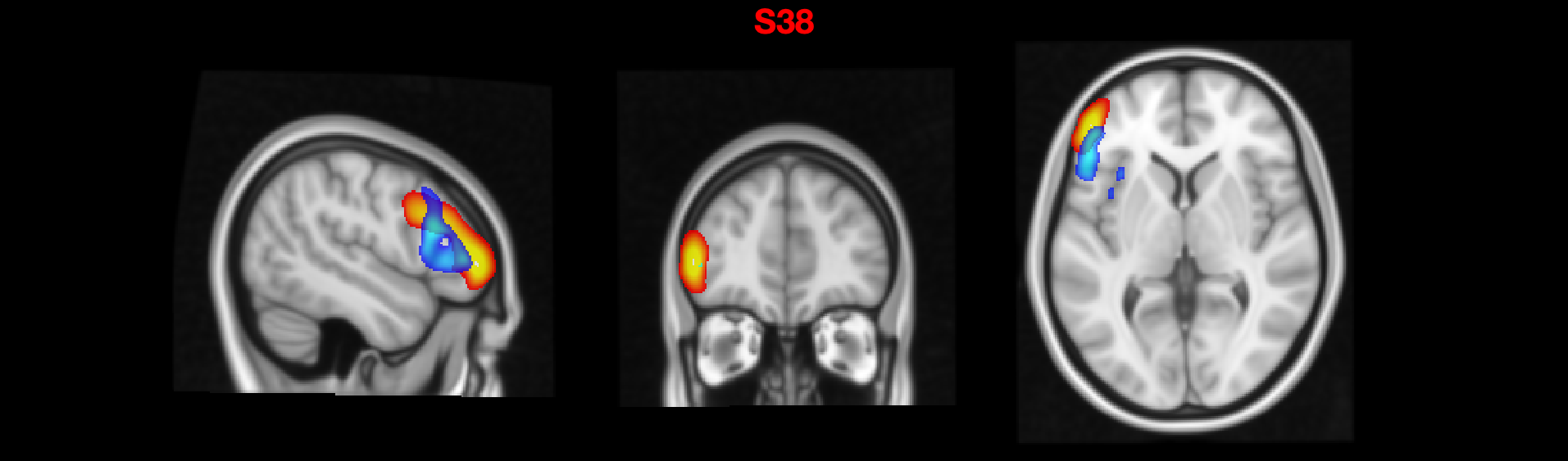

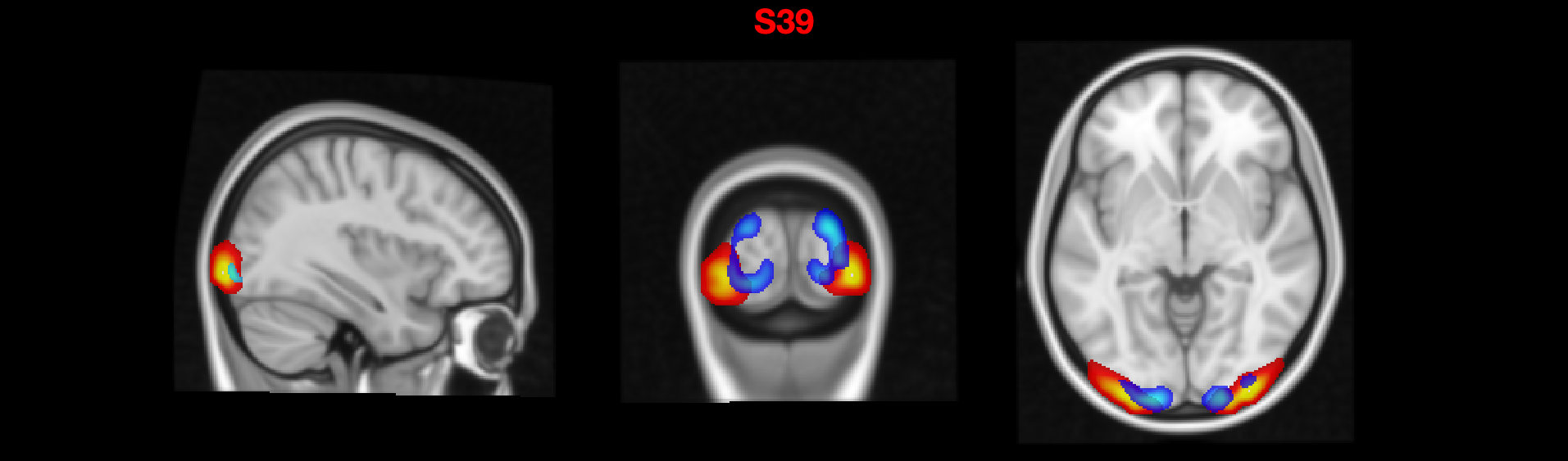

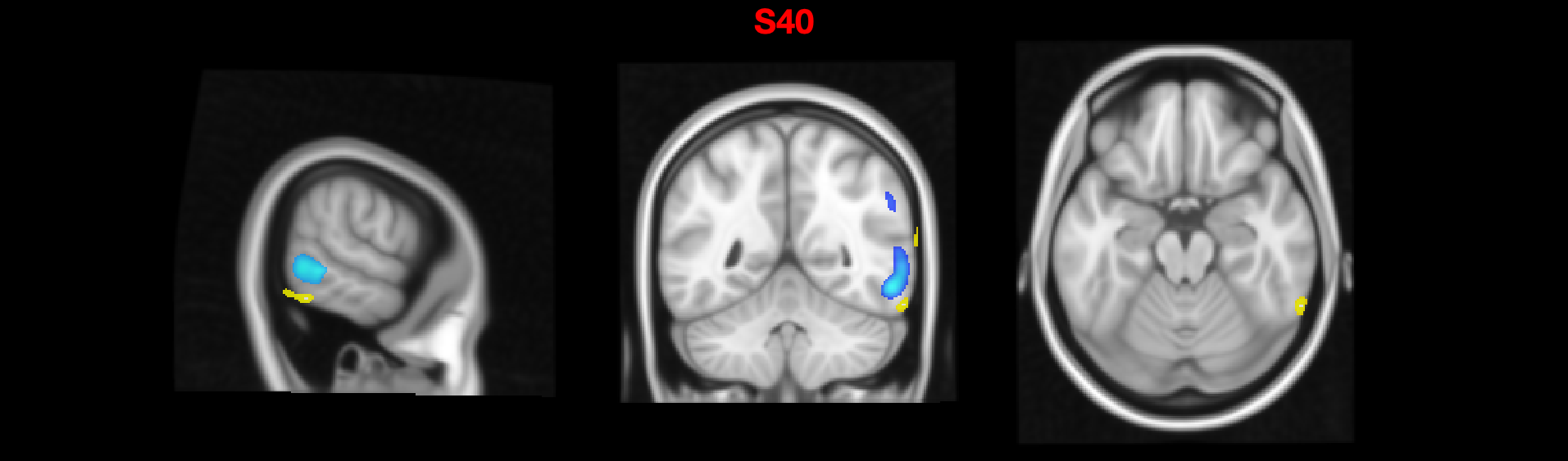

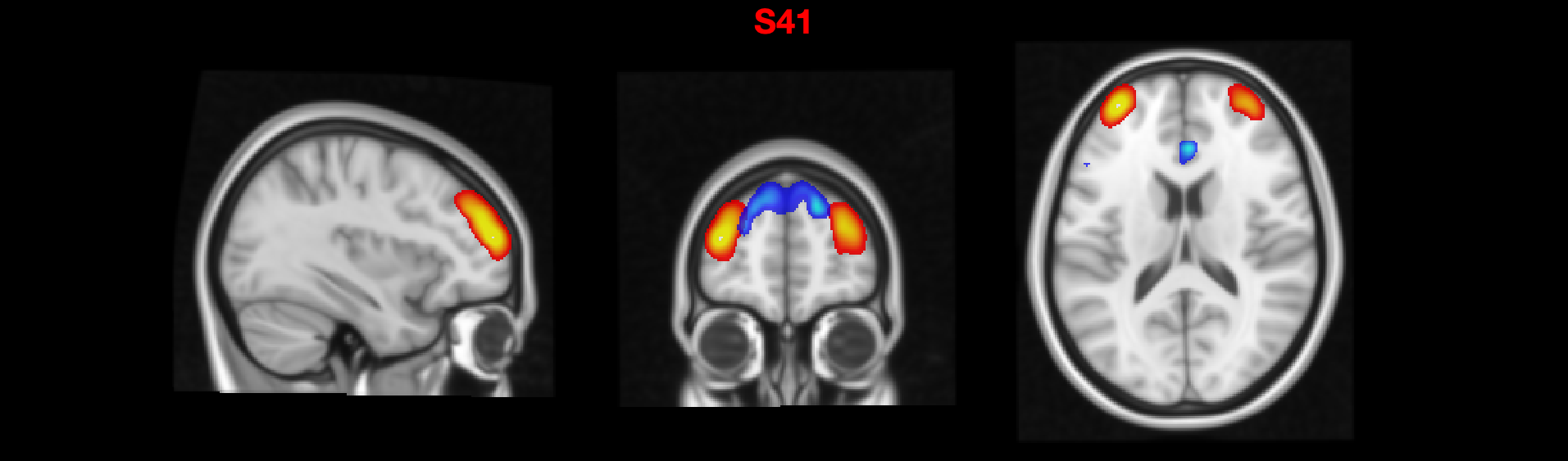

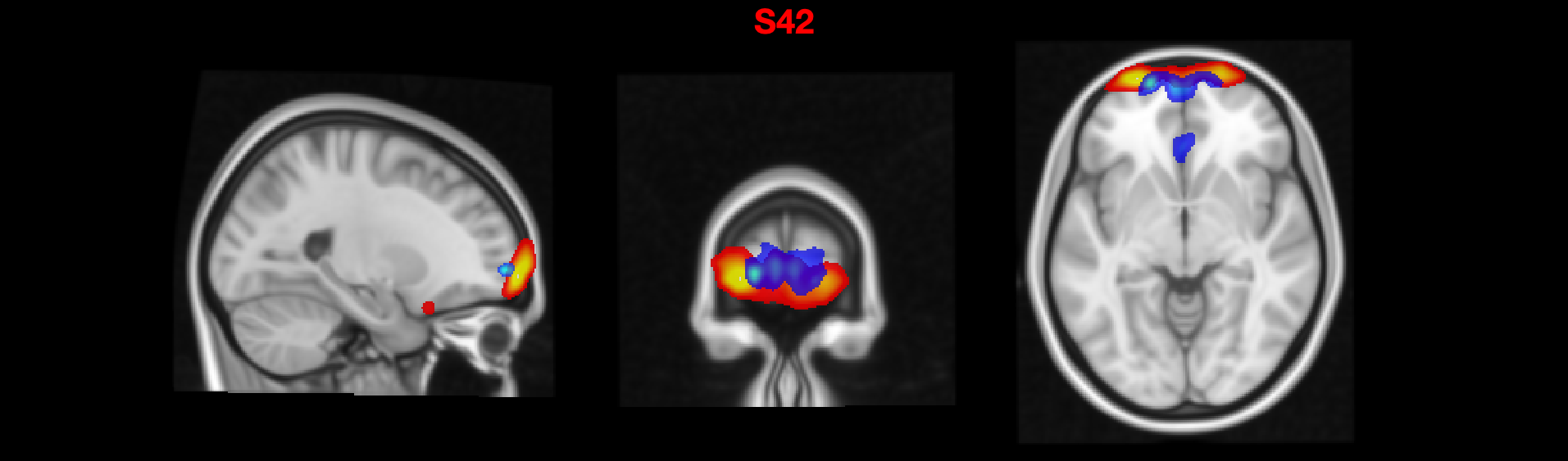

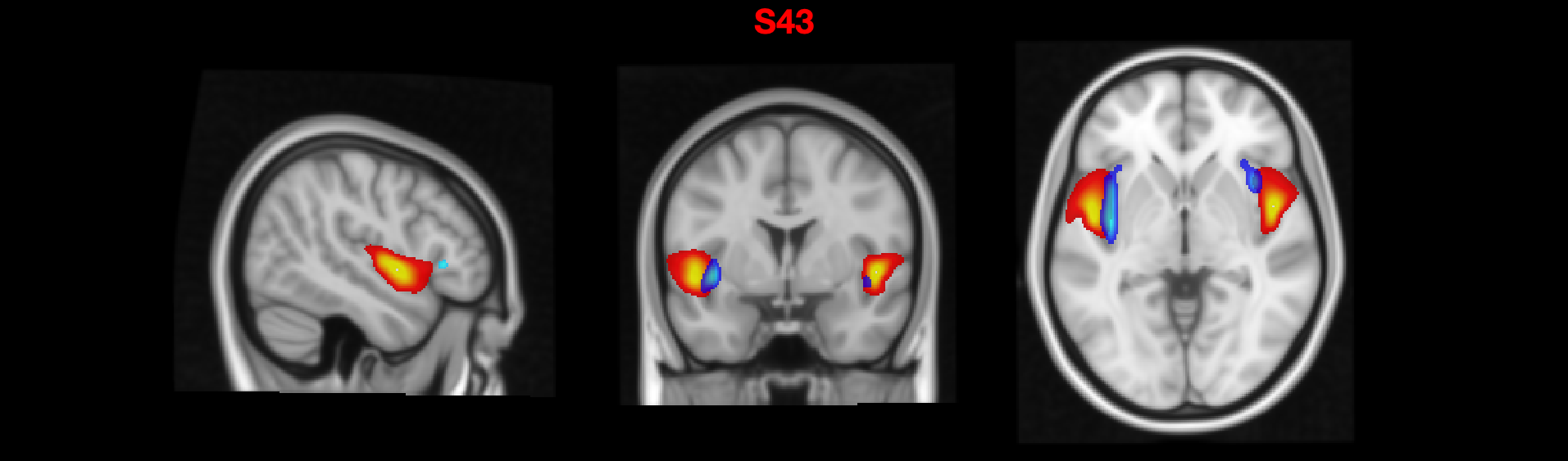

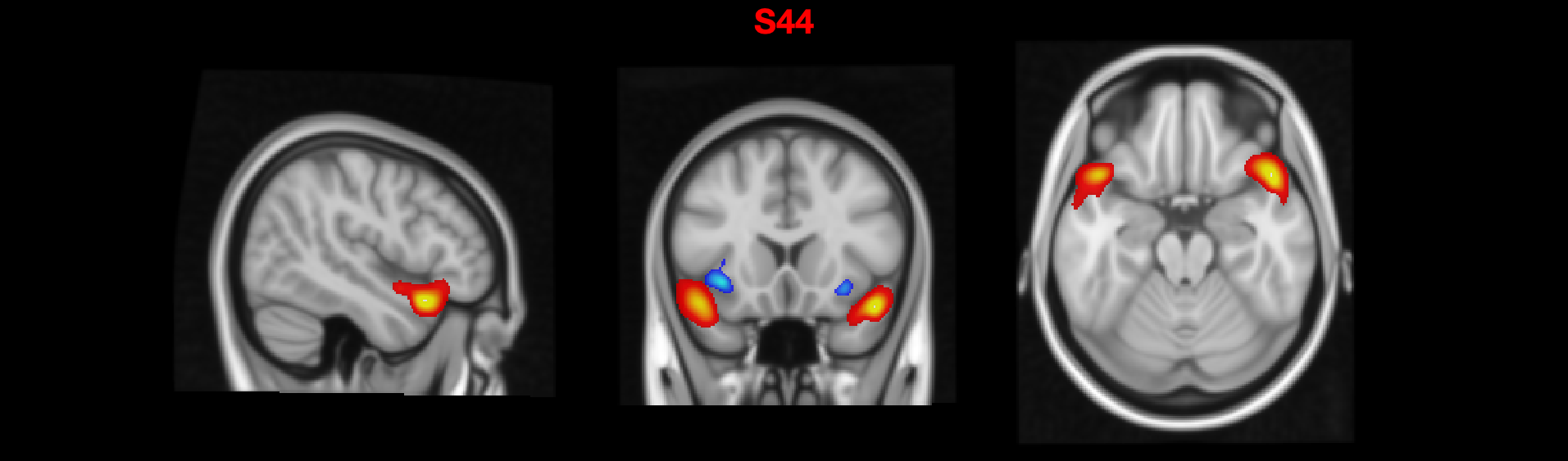

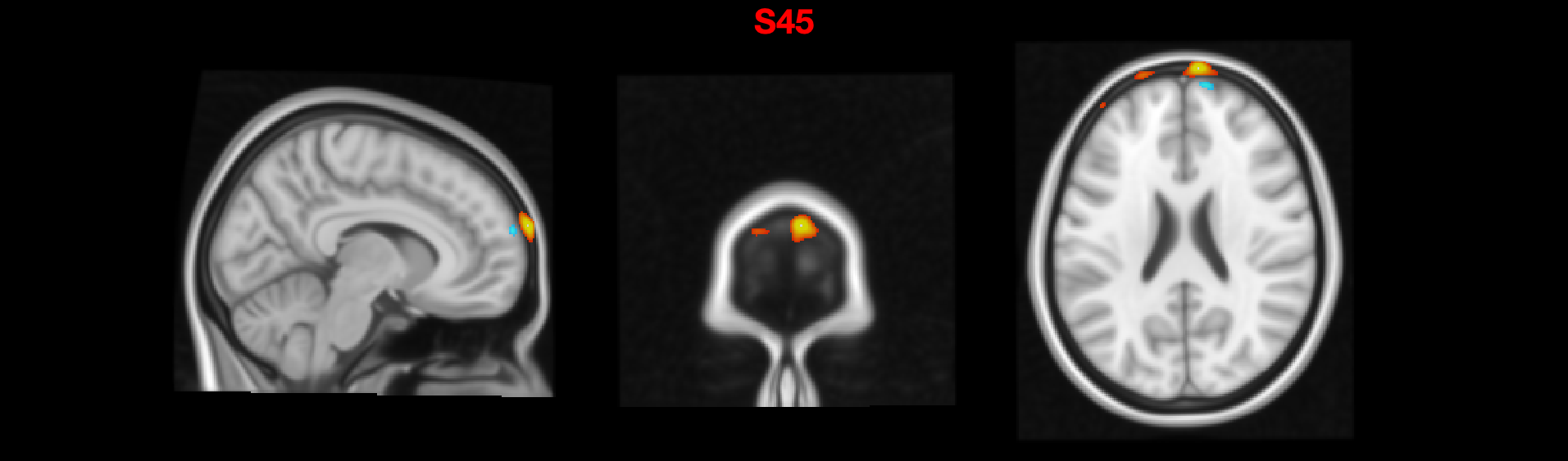

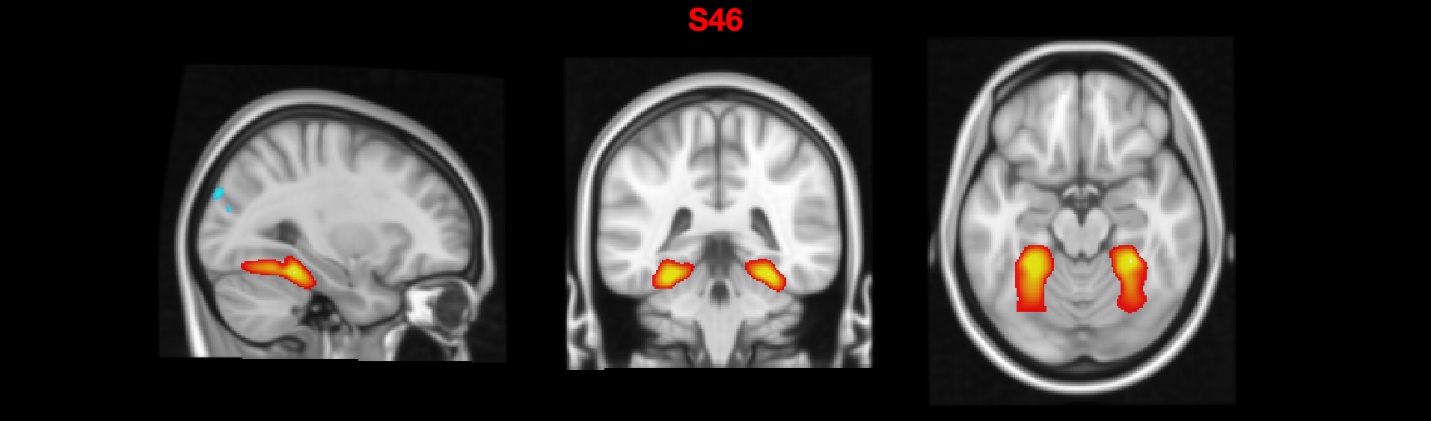

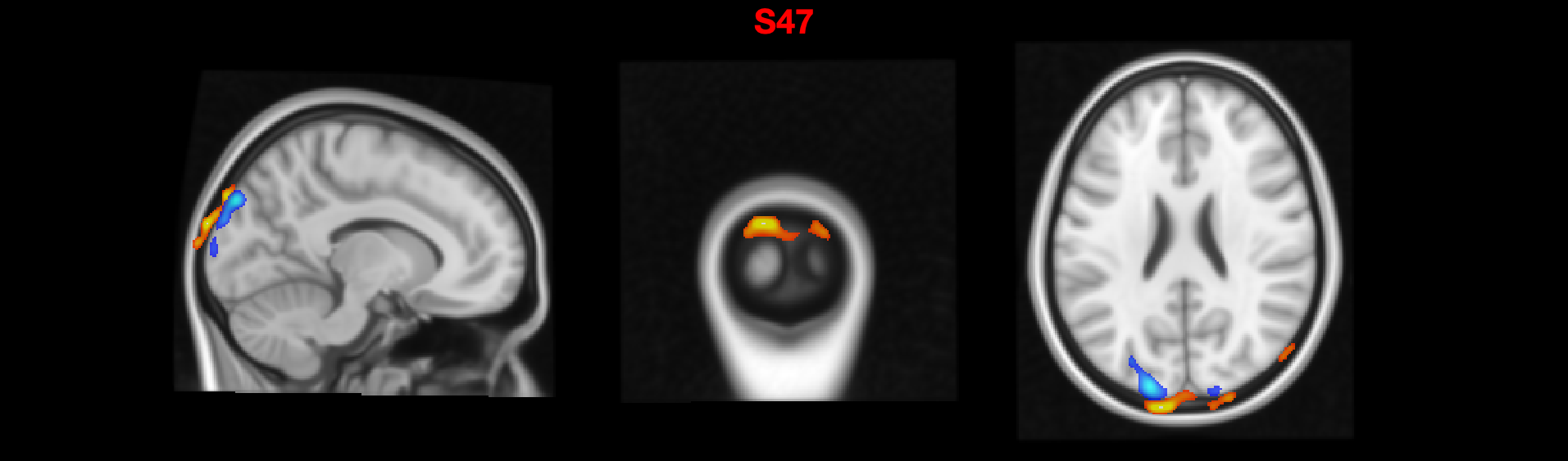

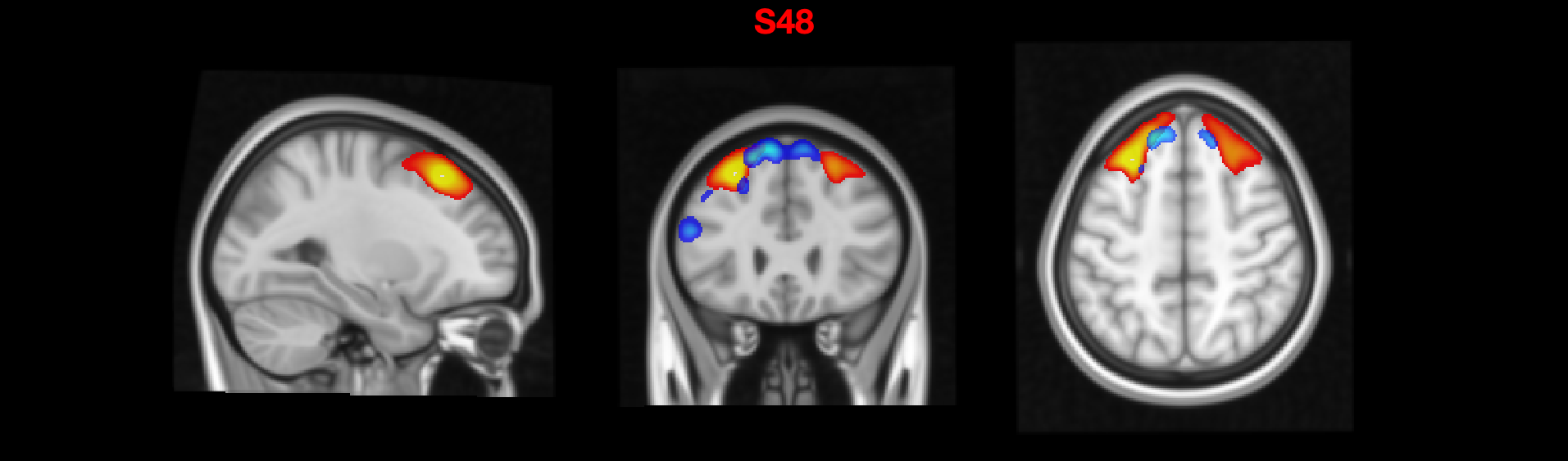

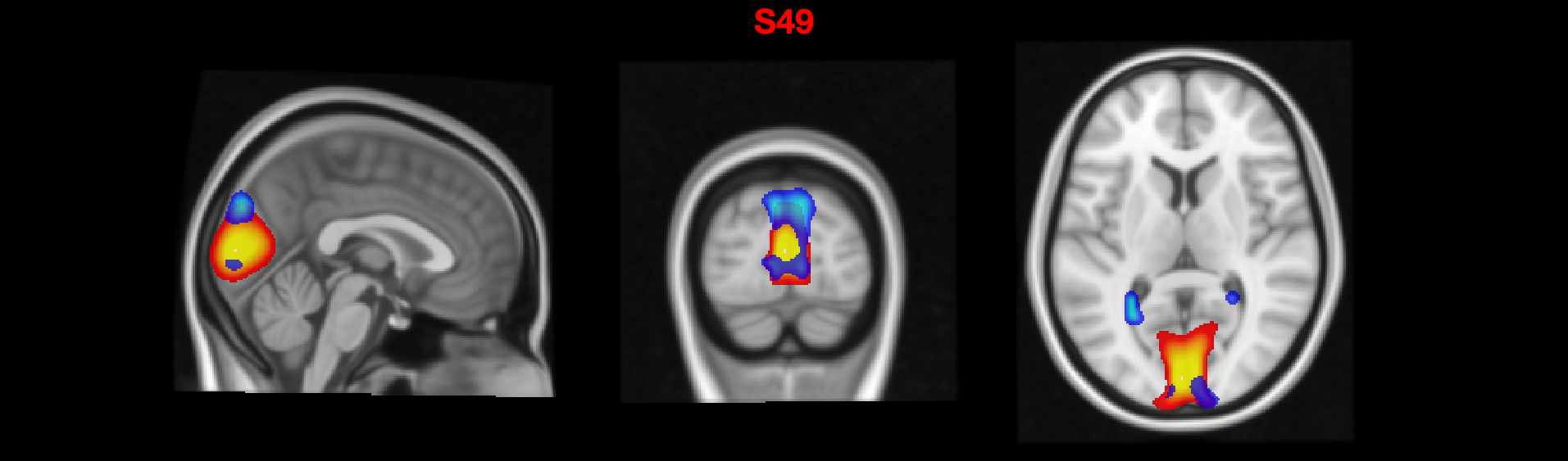

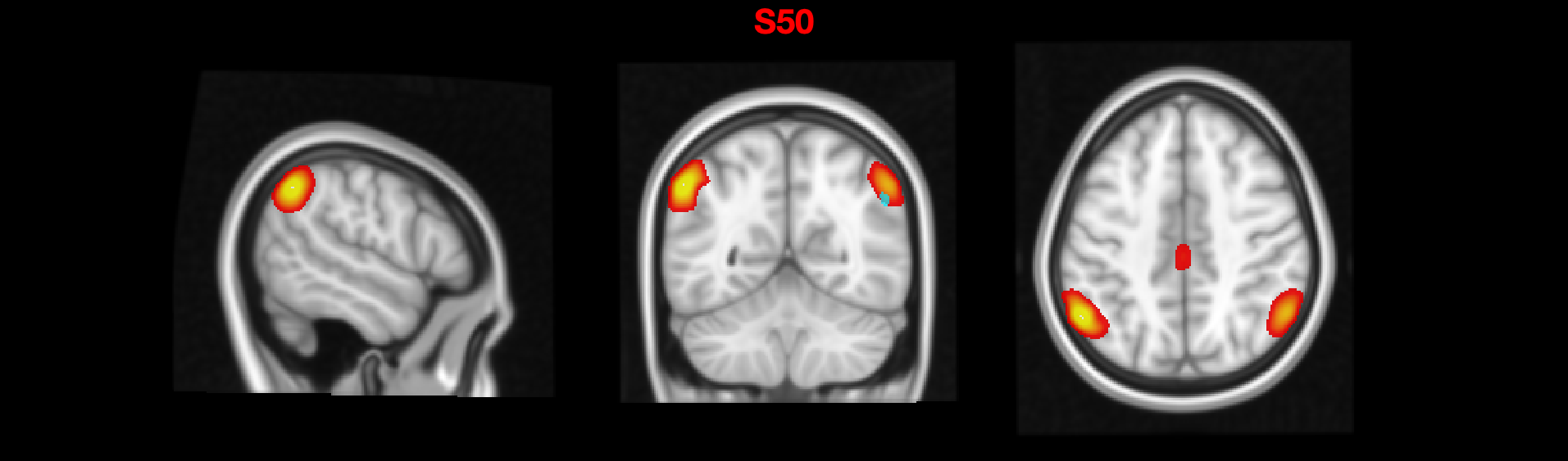

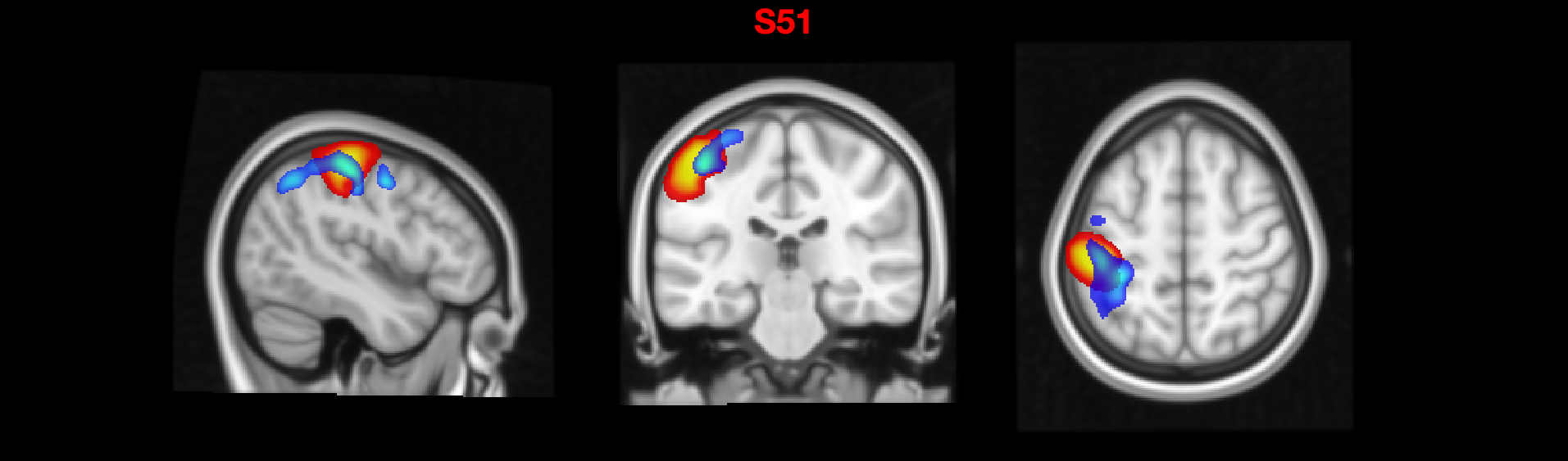

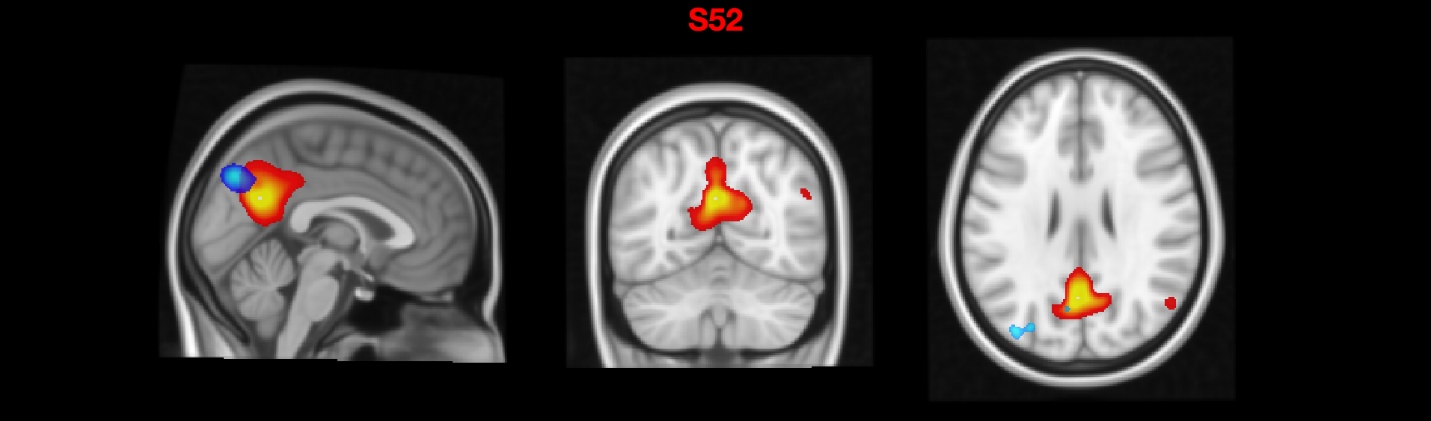

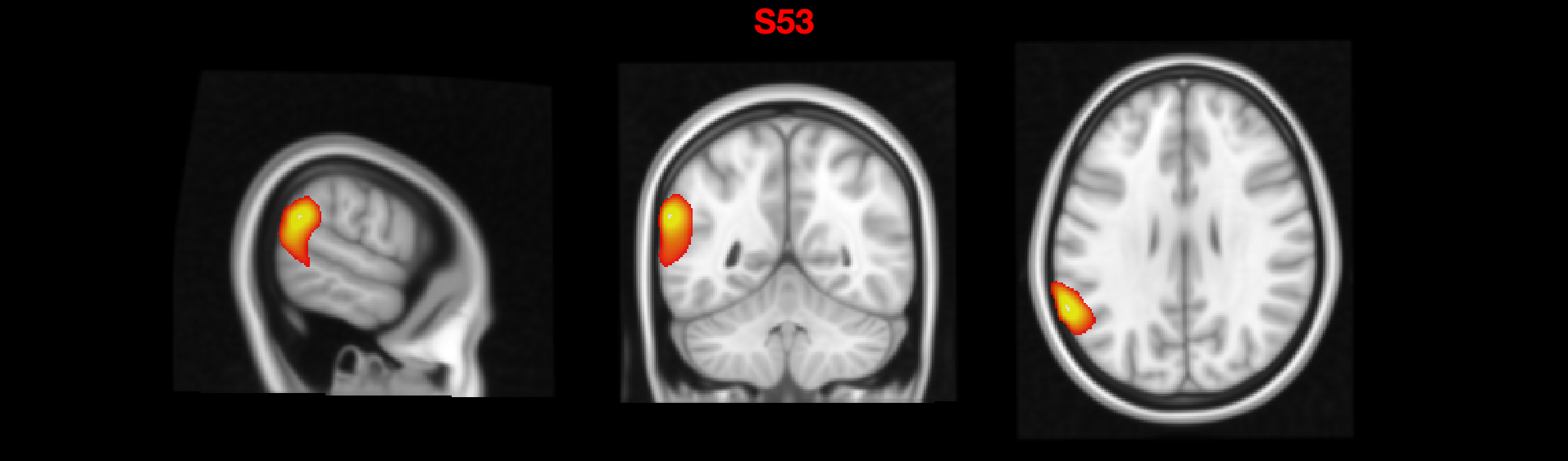

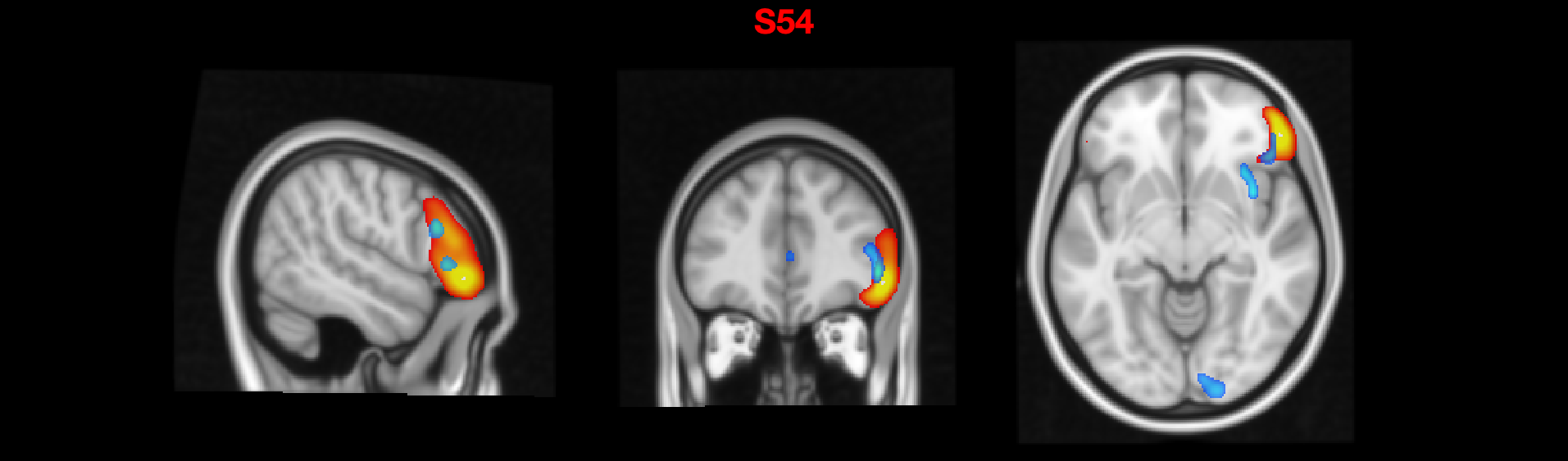

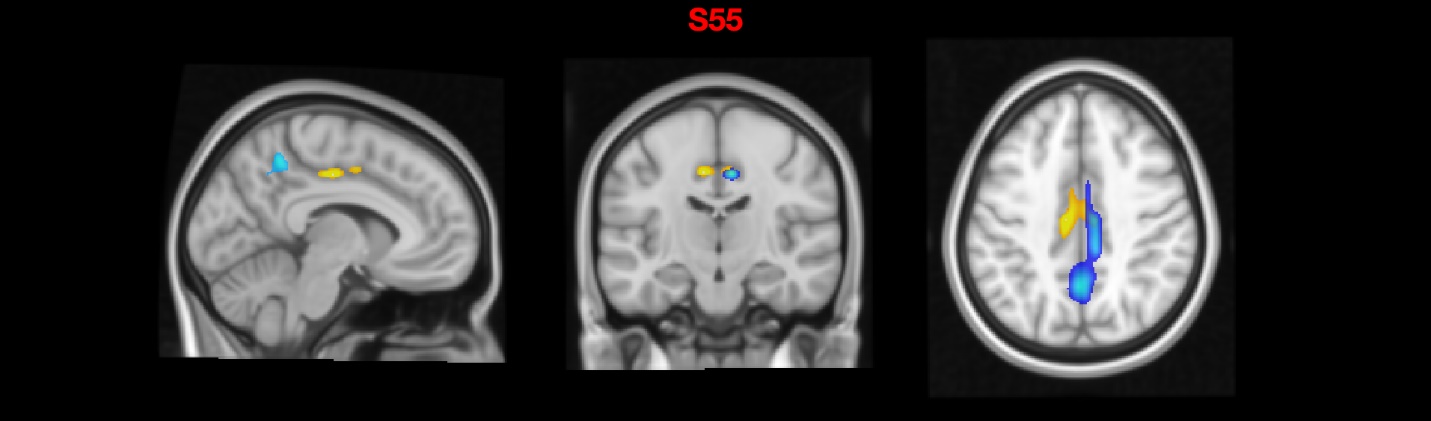

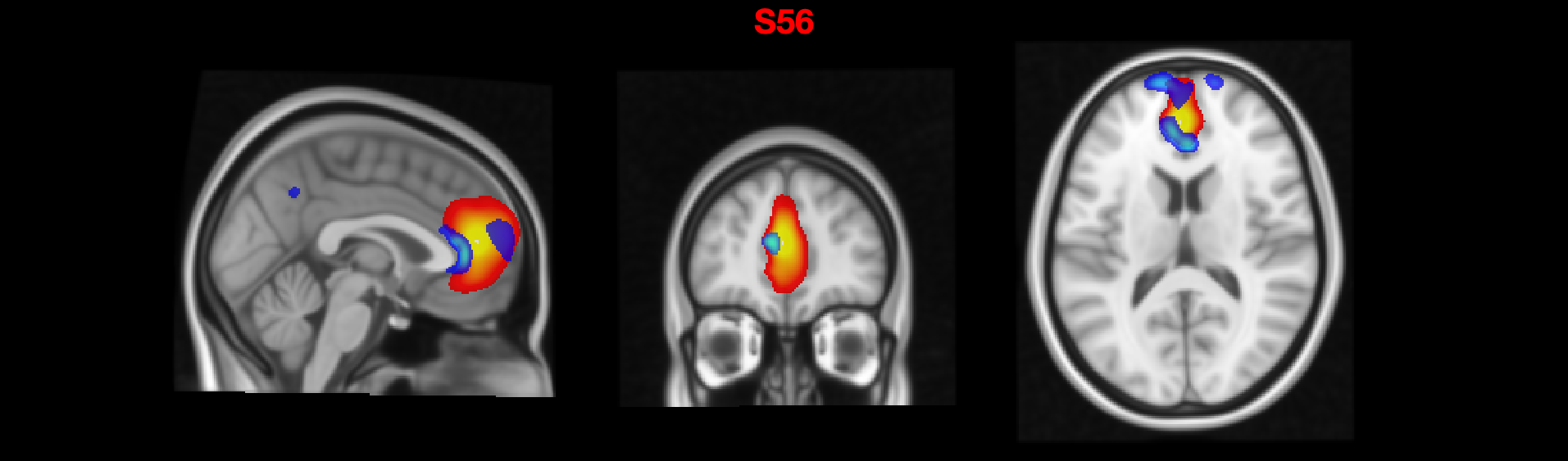

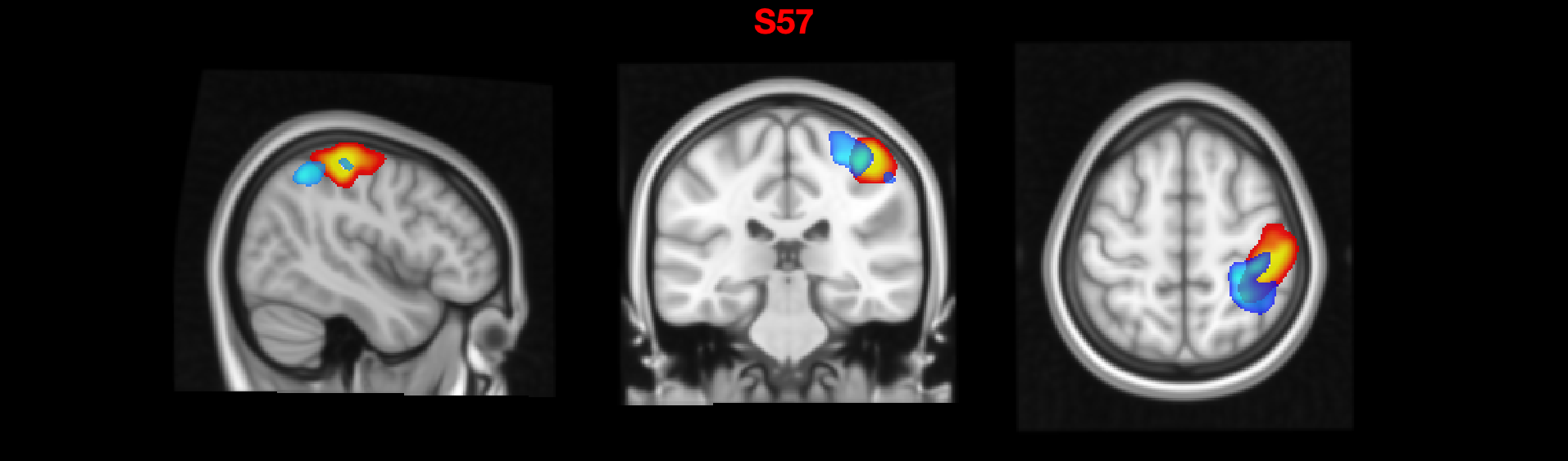

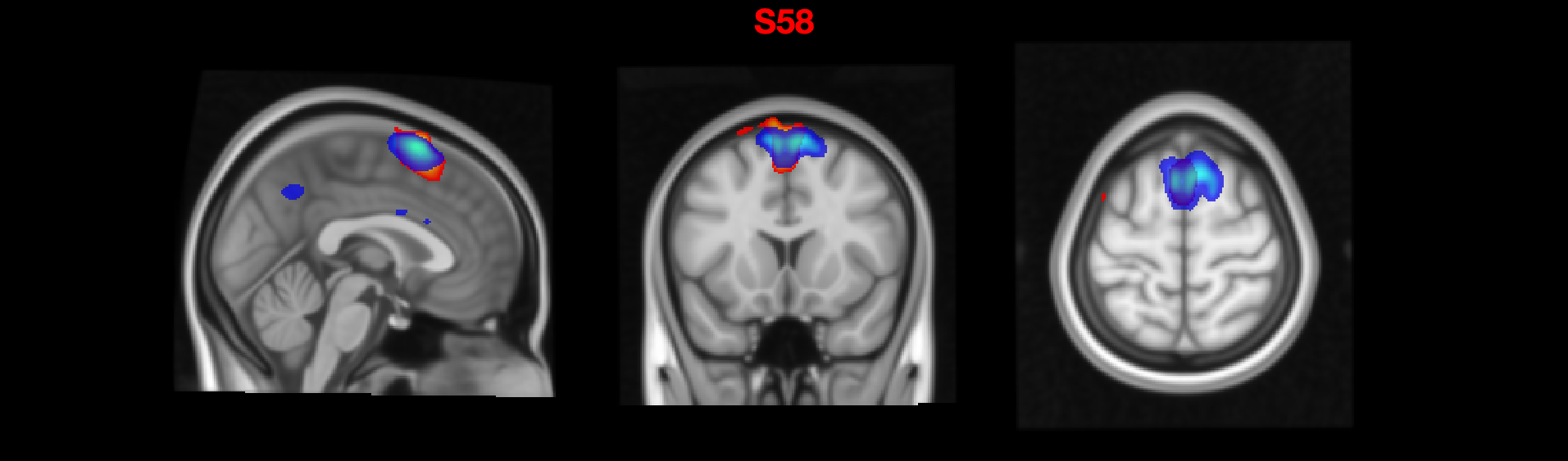

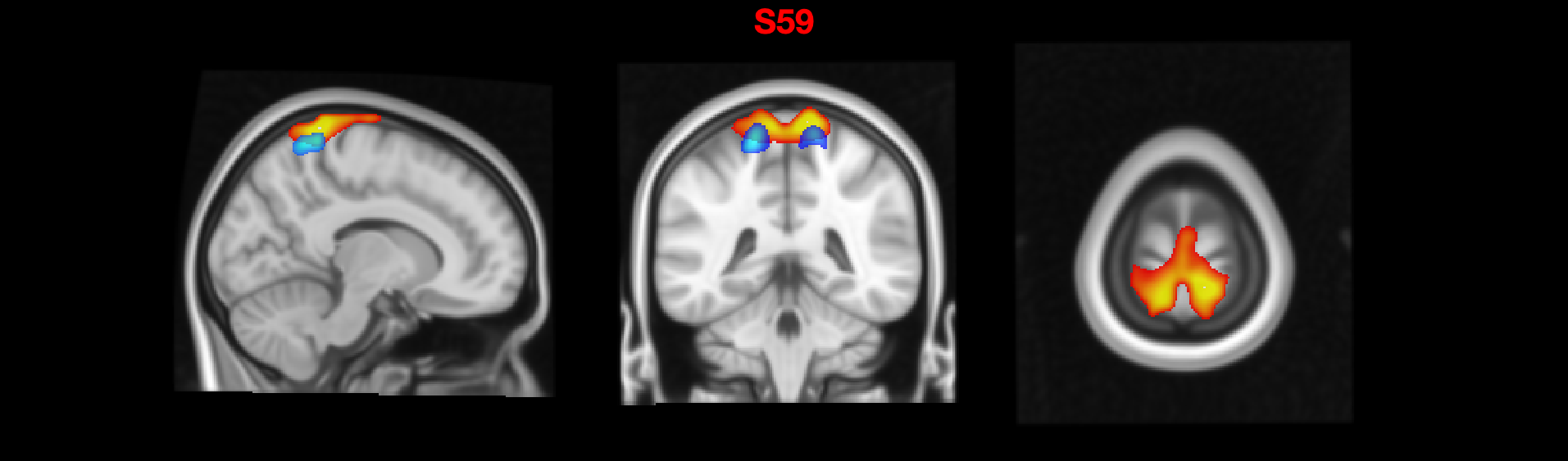

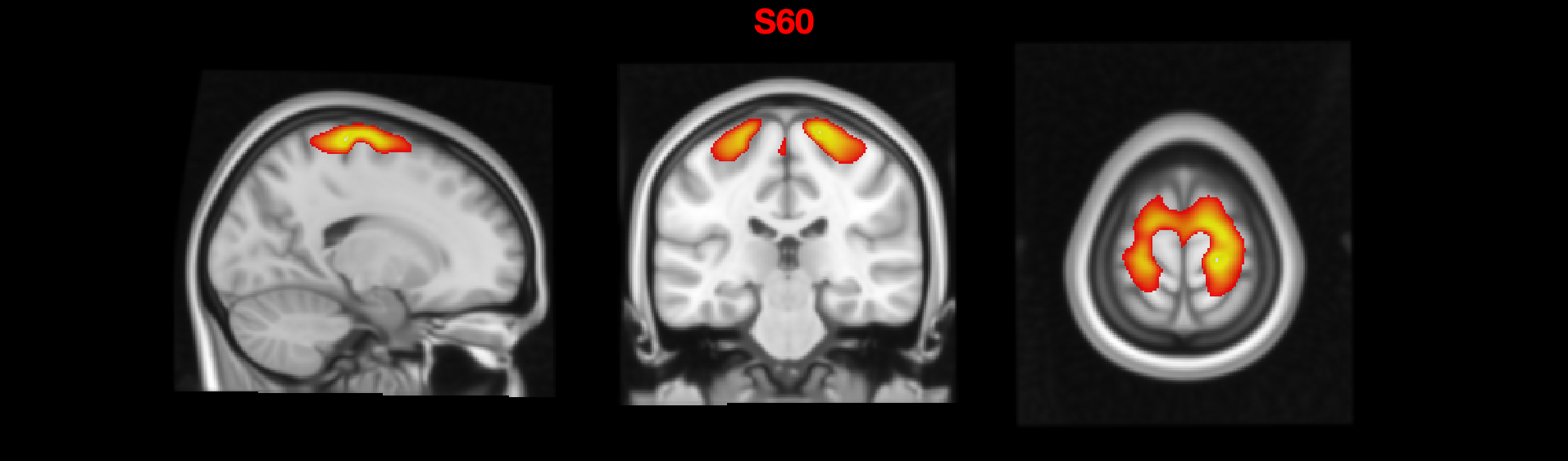


1. Joint cmICA *R* maps of FCN (red) and WMT (blue)


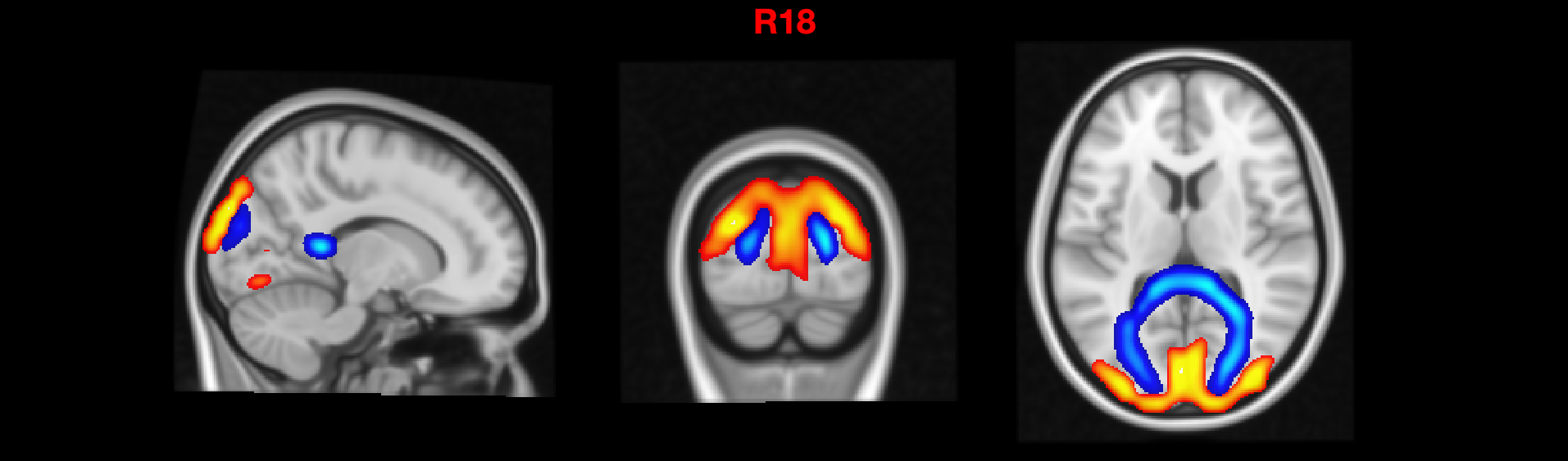

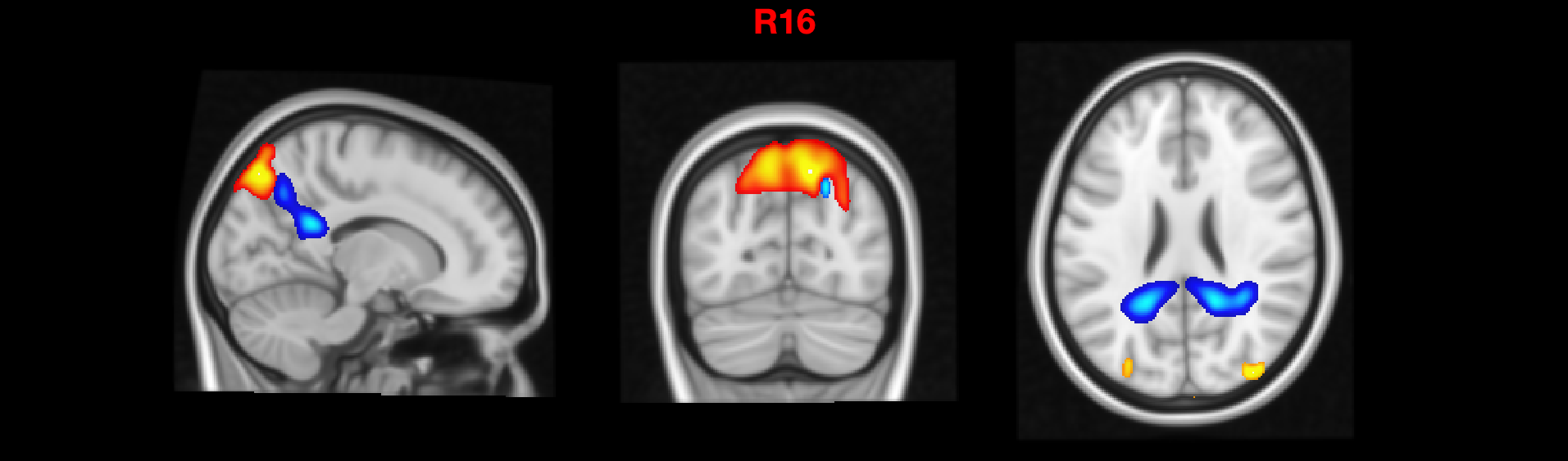

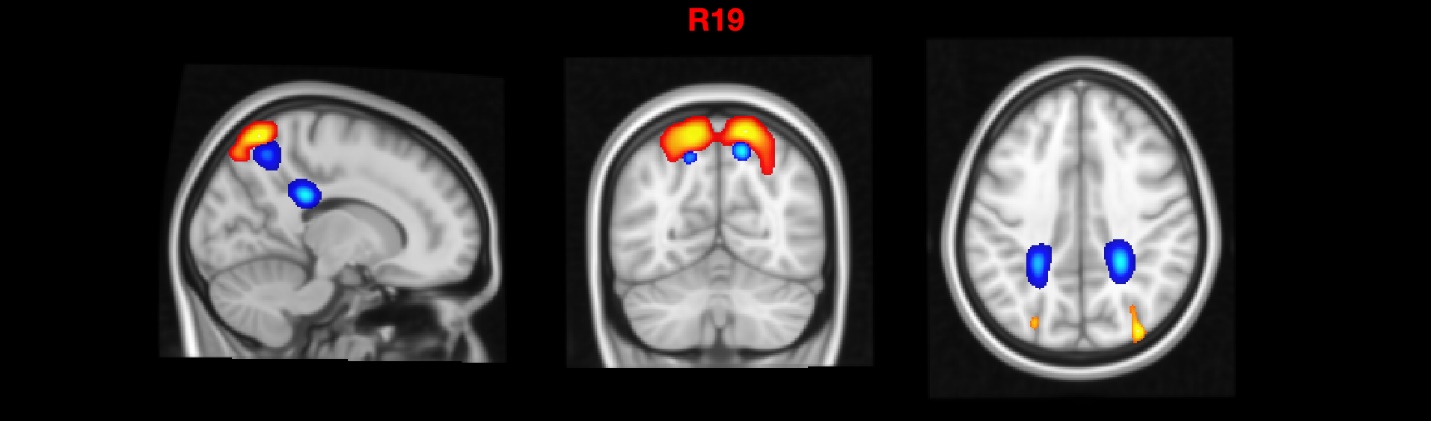

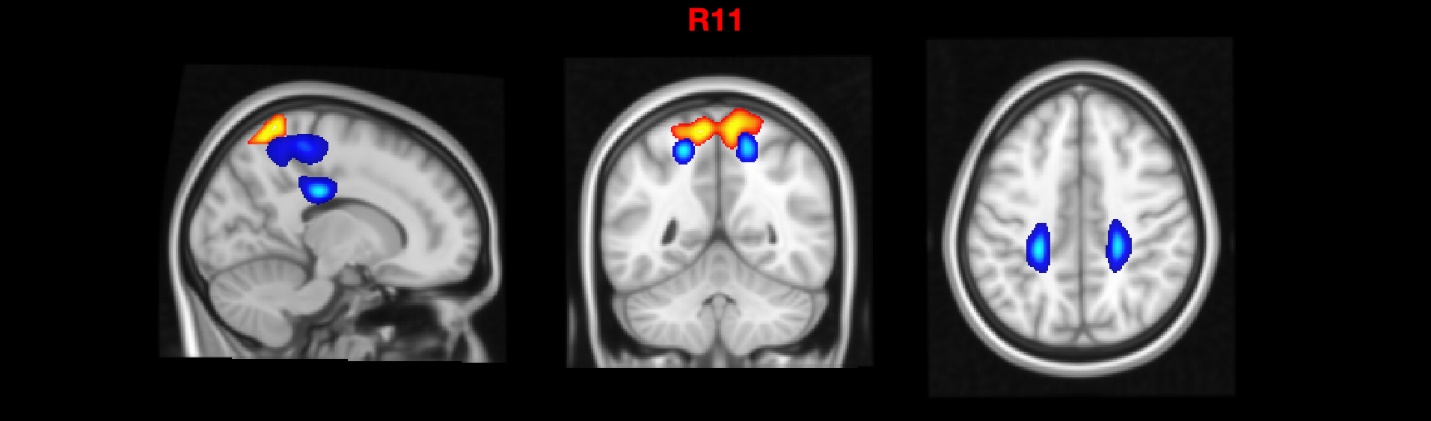

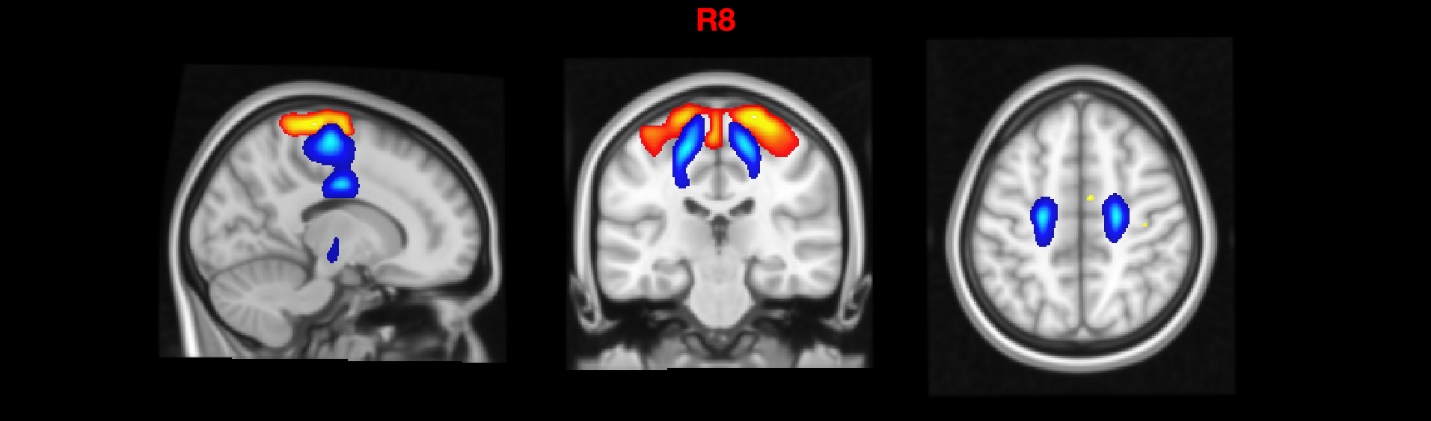

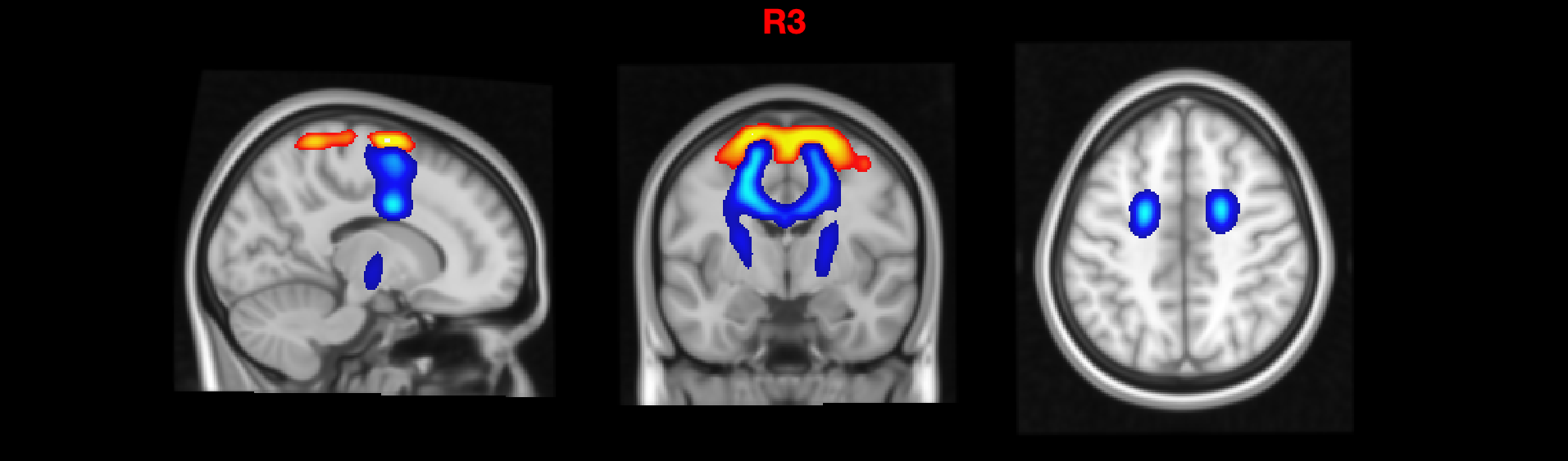

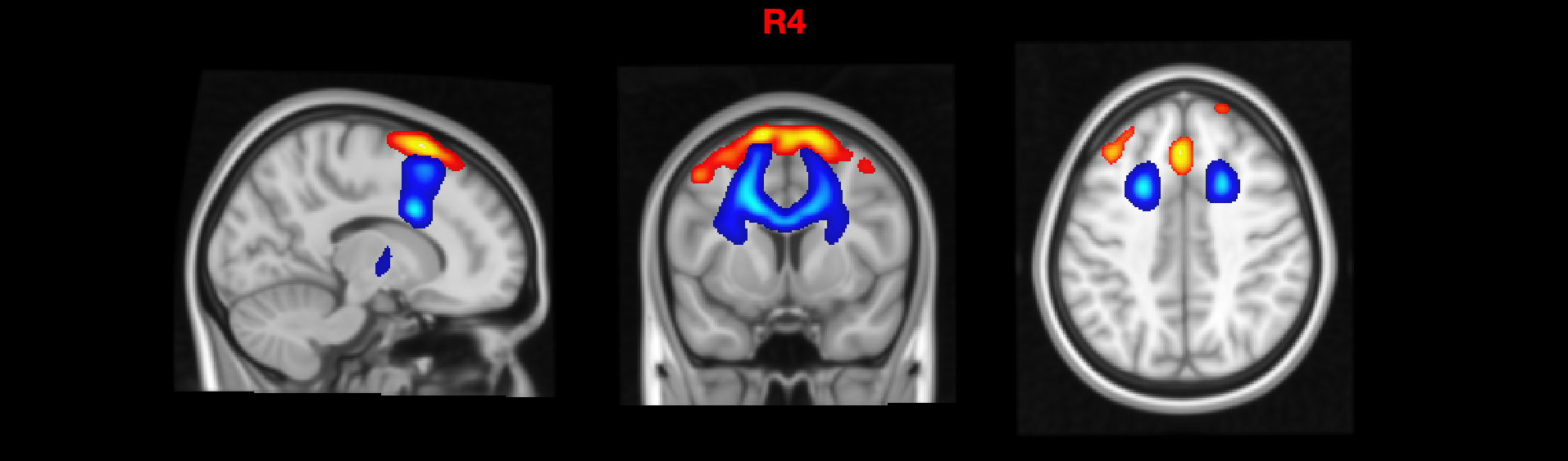

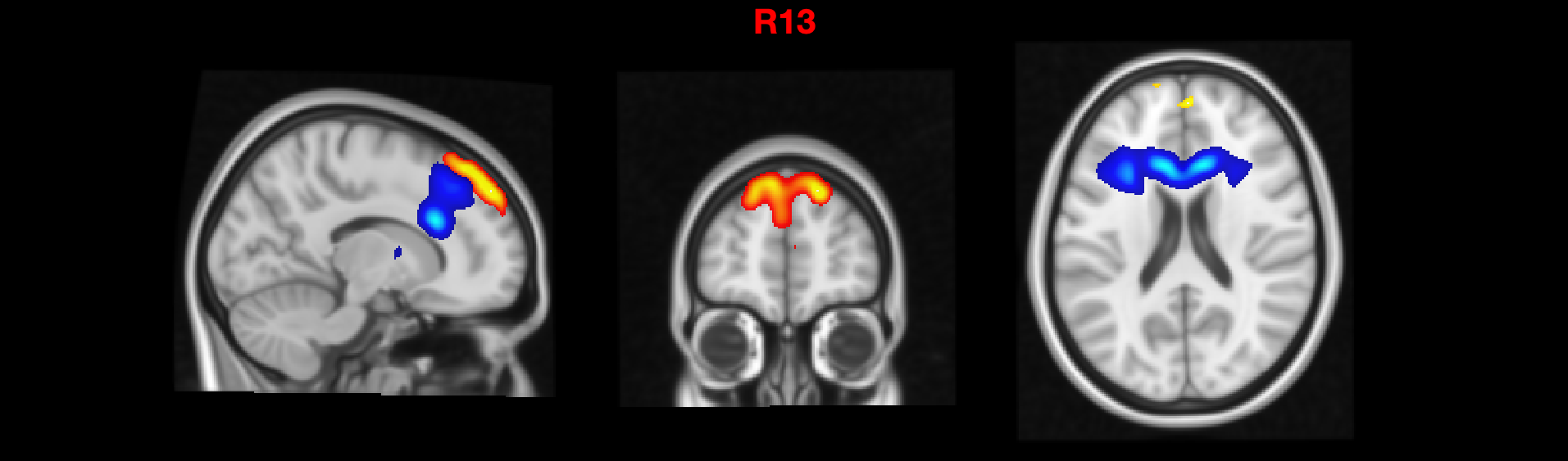

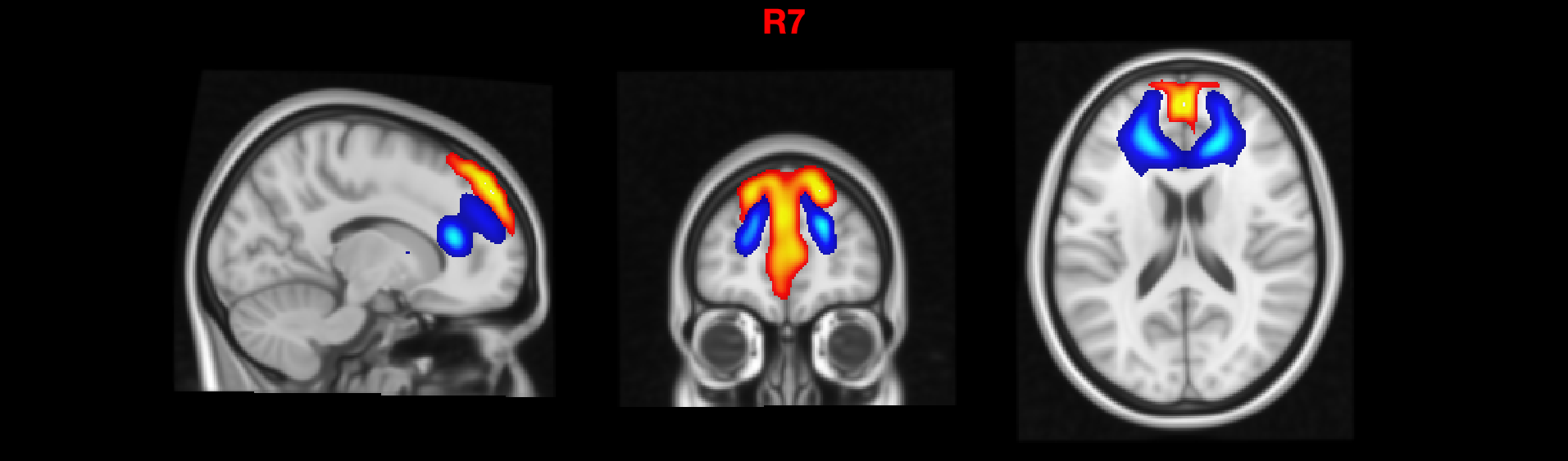

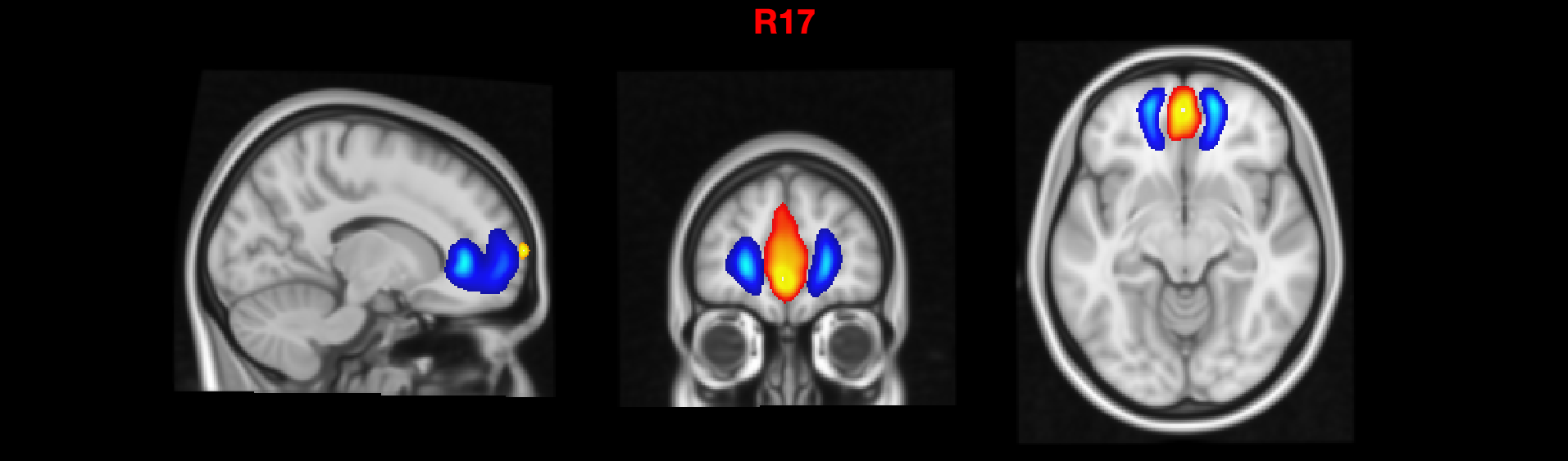

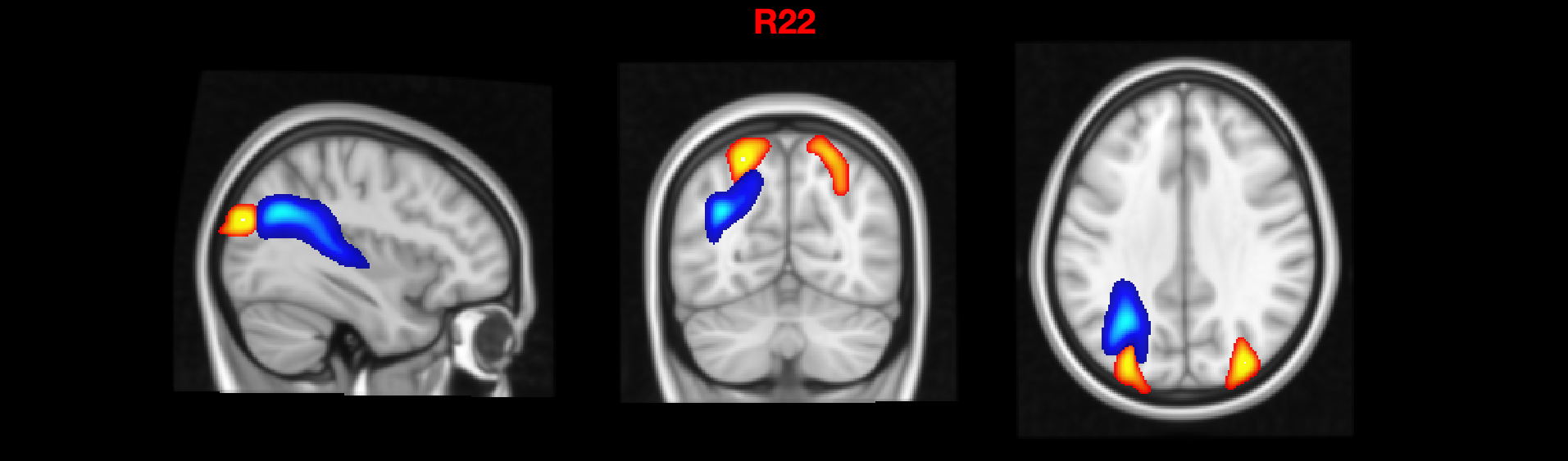

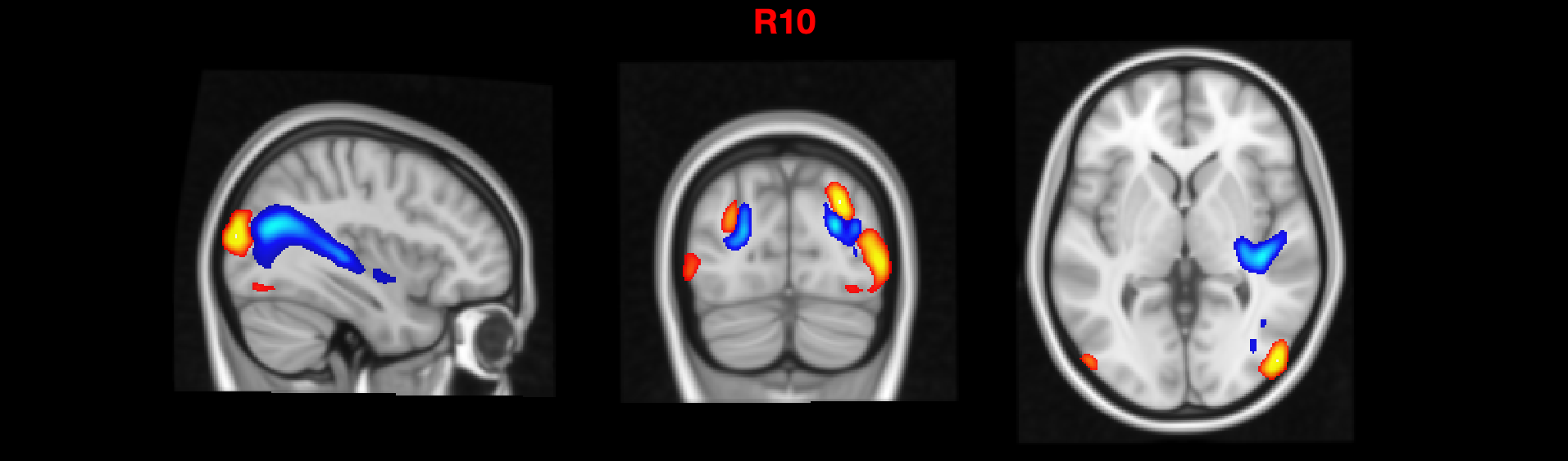

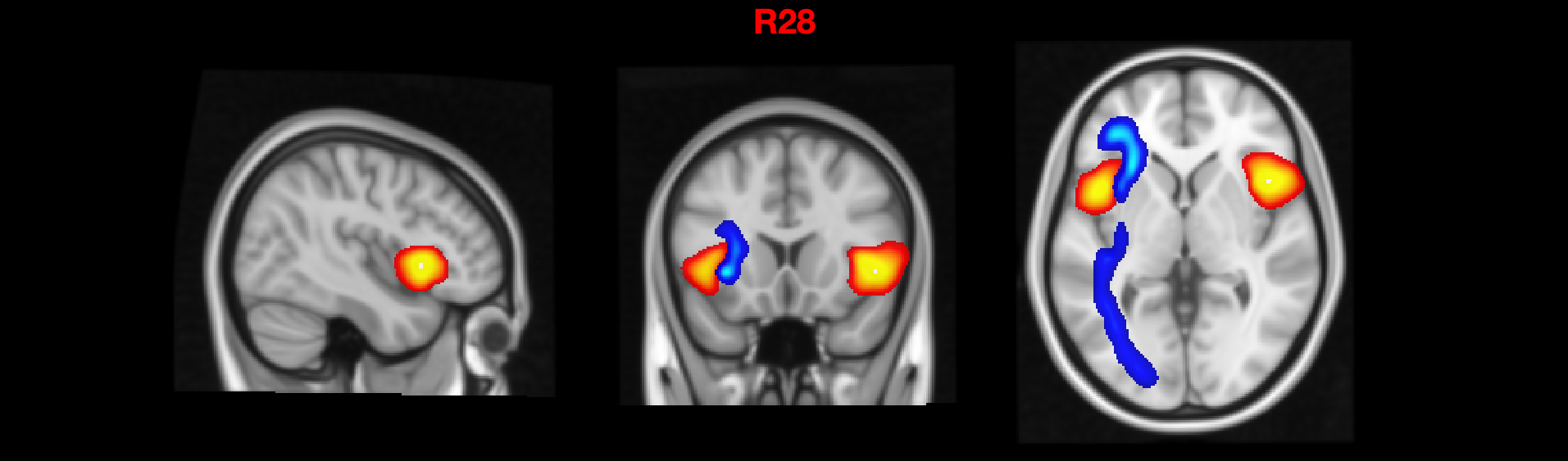

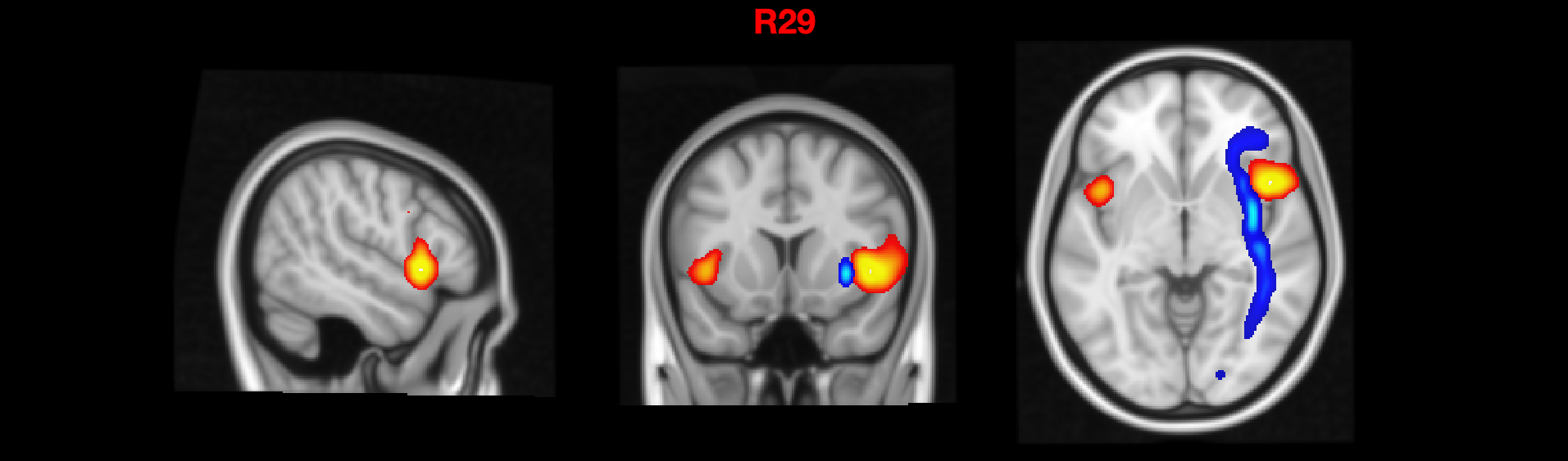

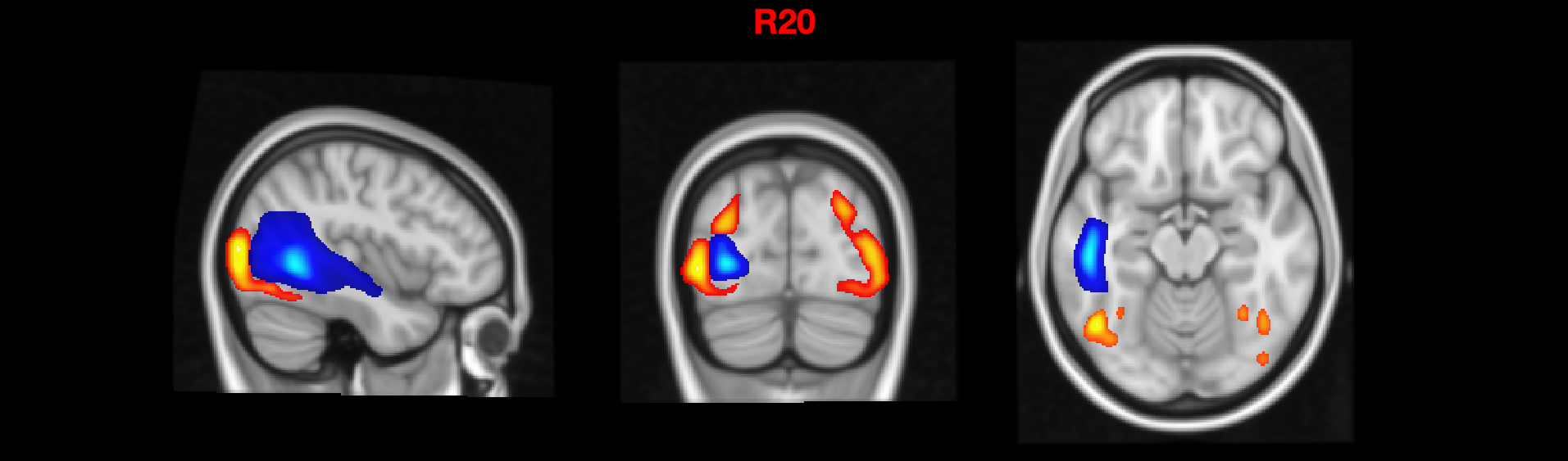

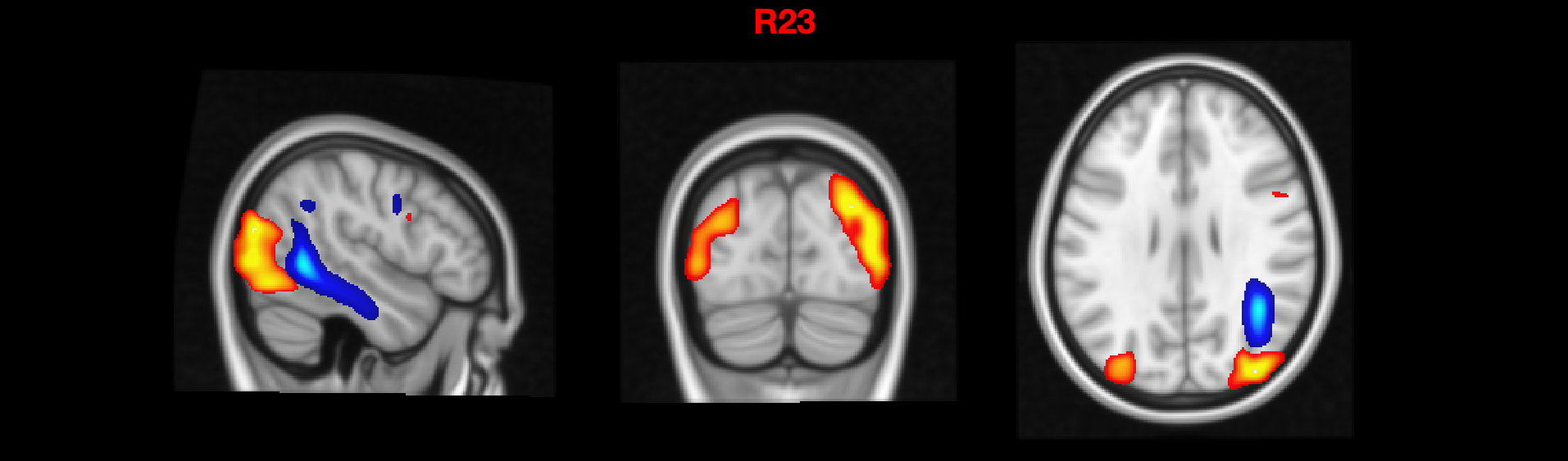

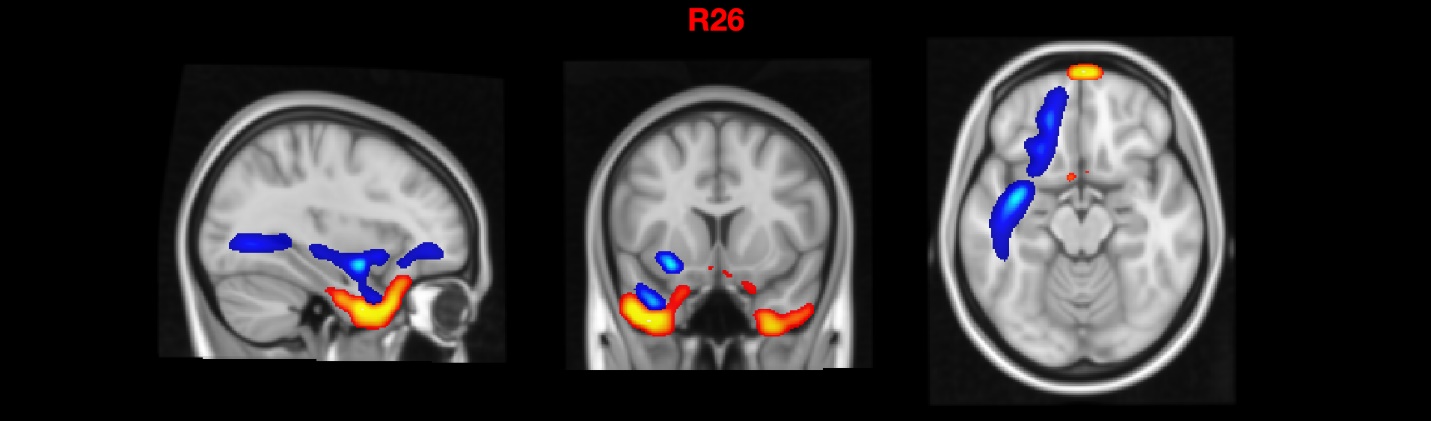

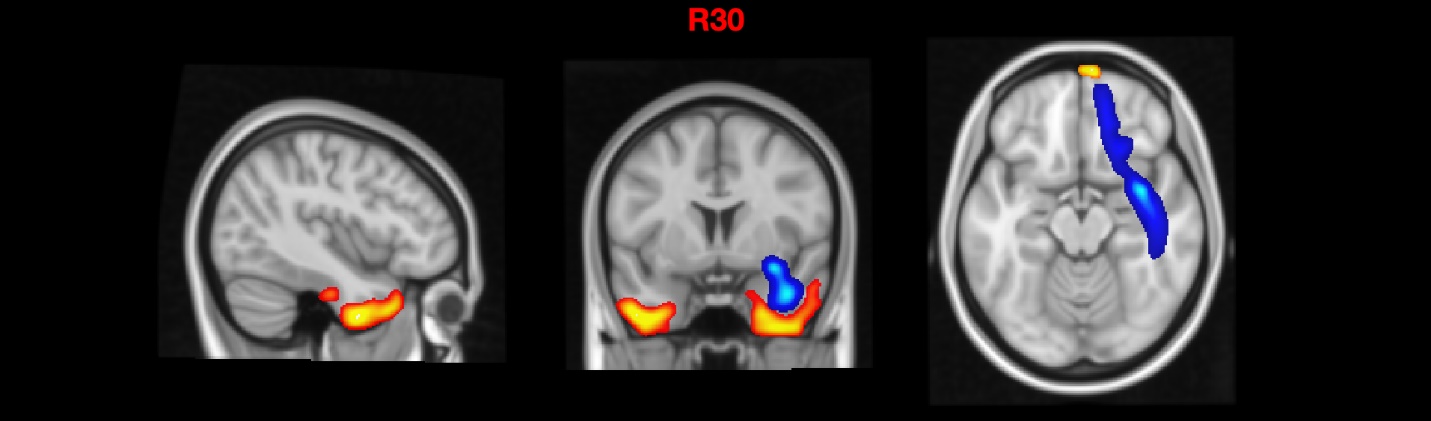

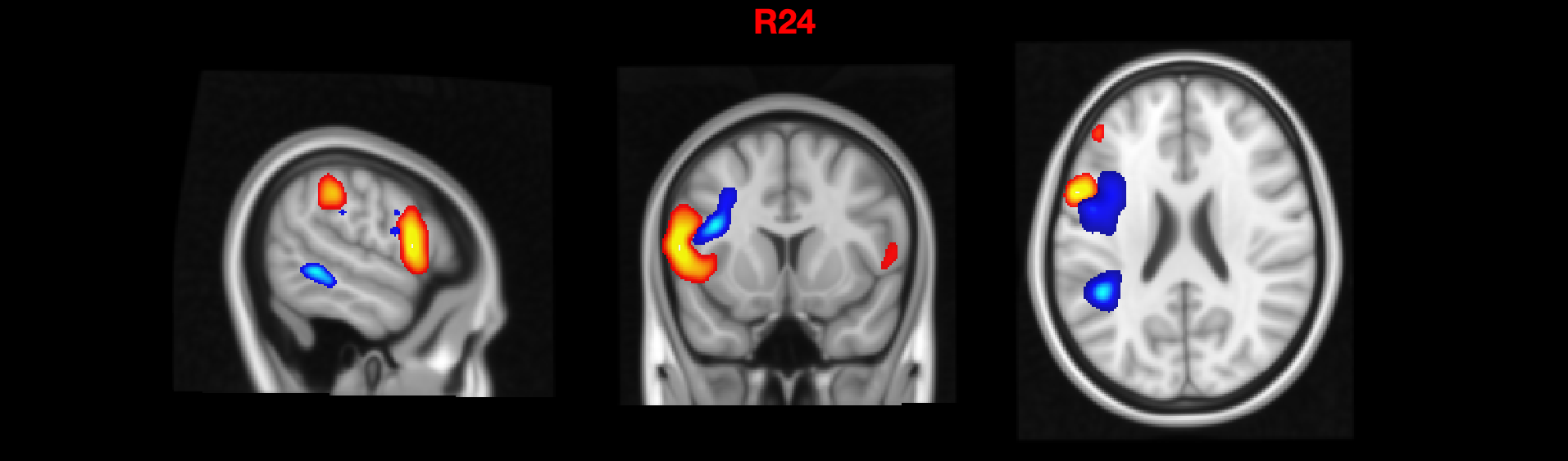

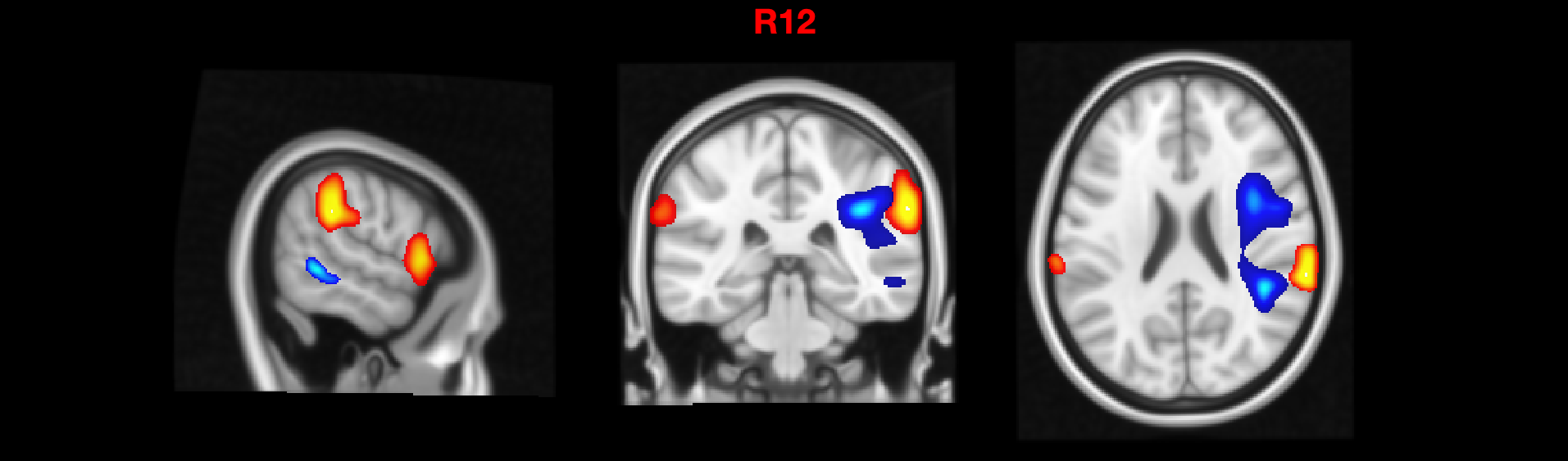

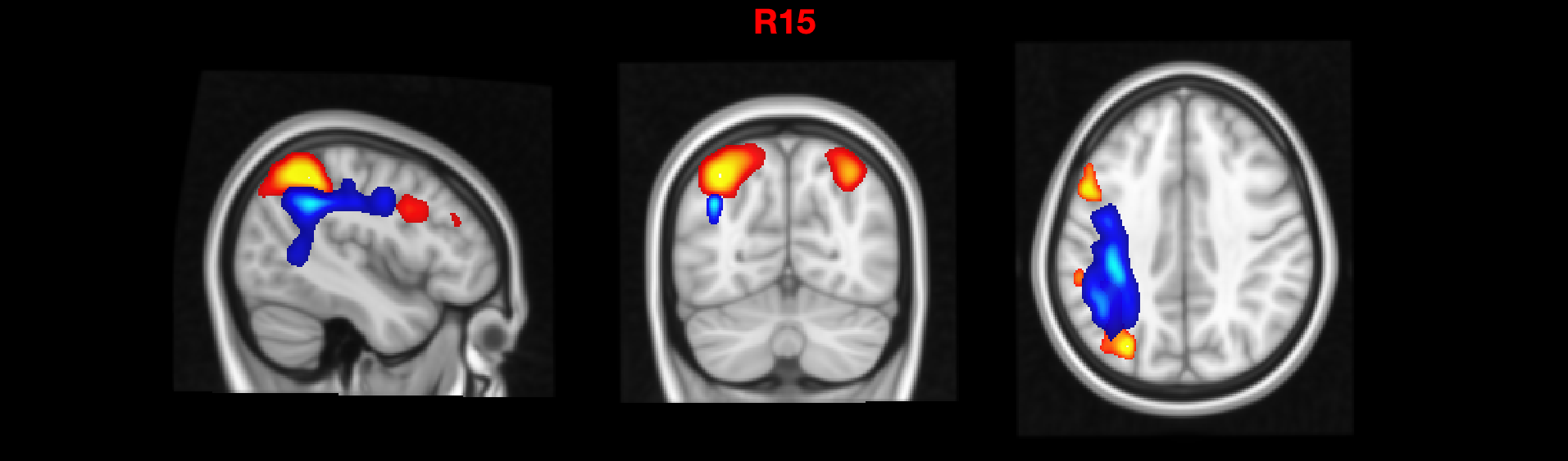

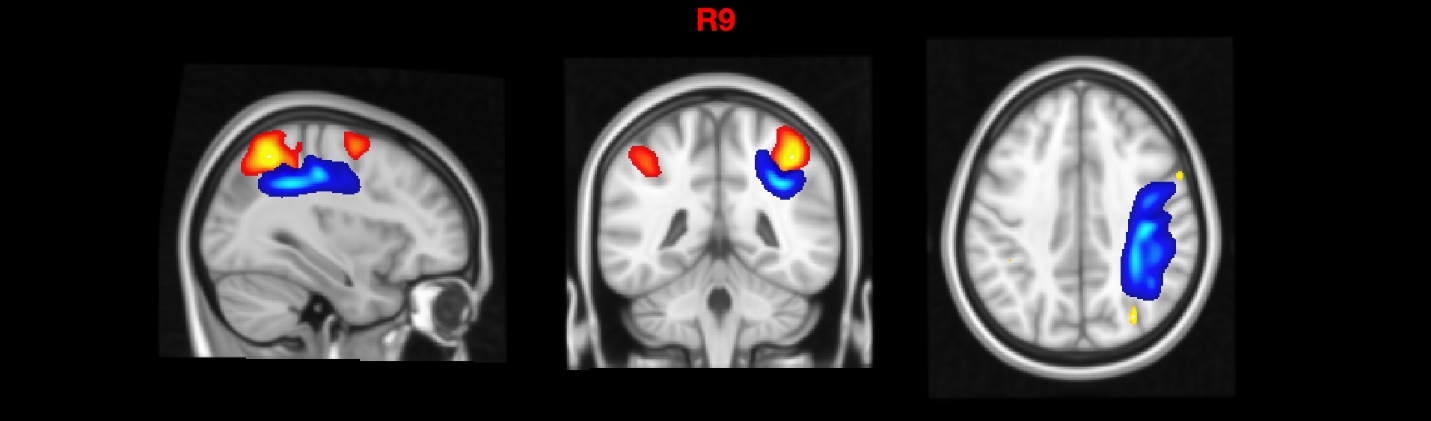

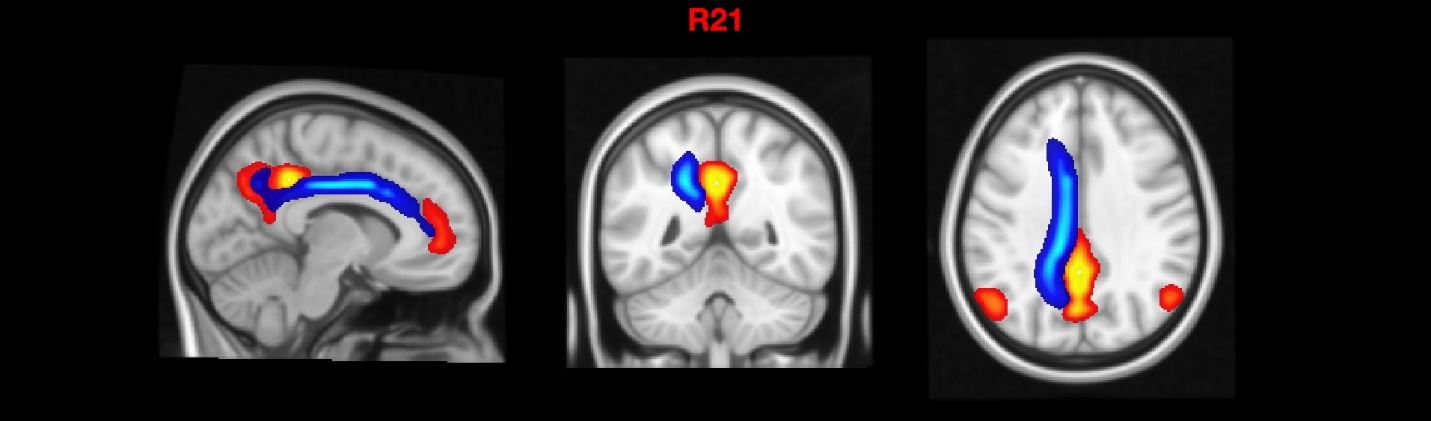

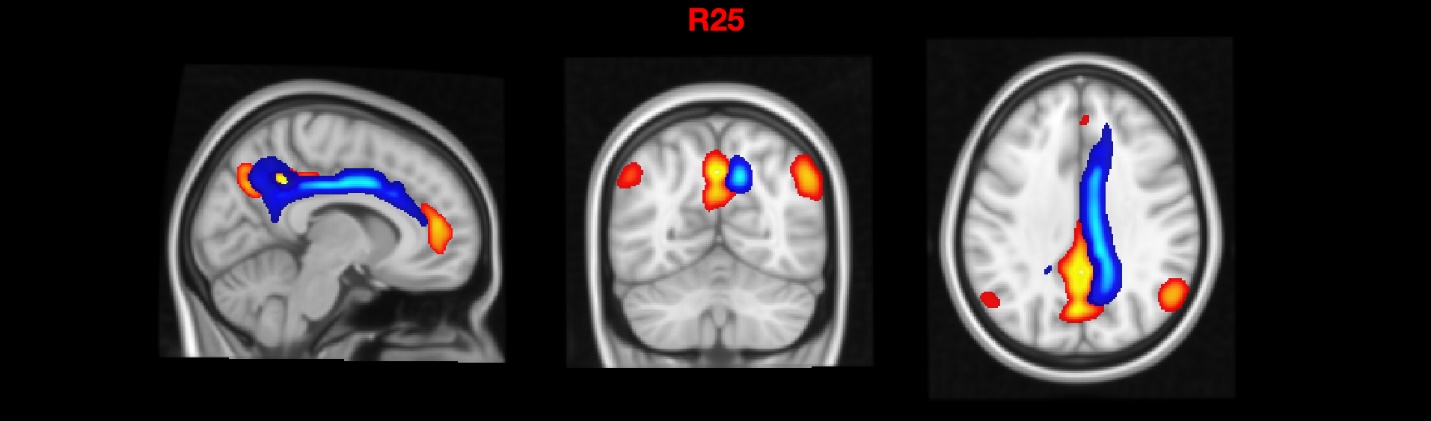

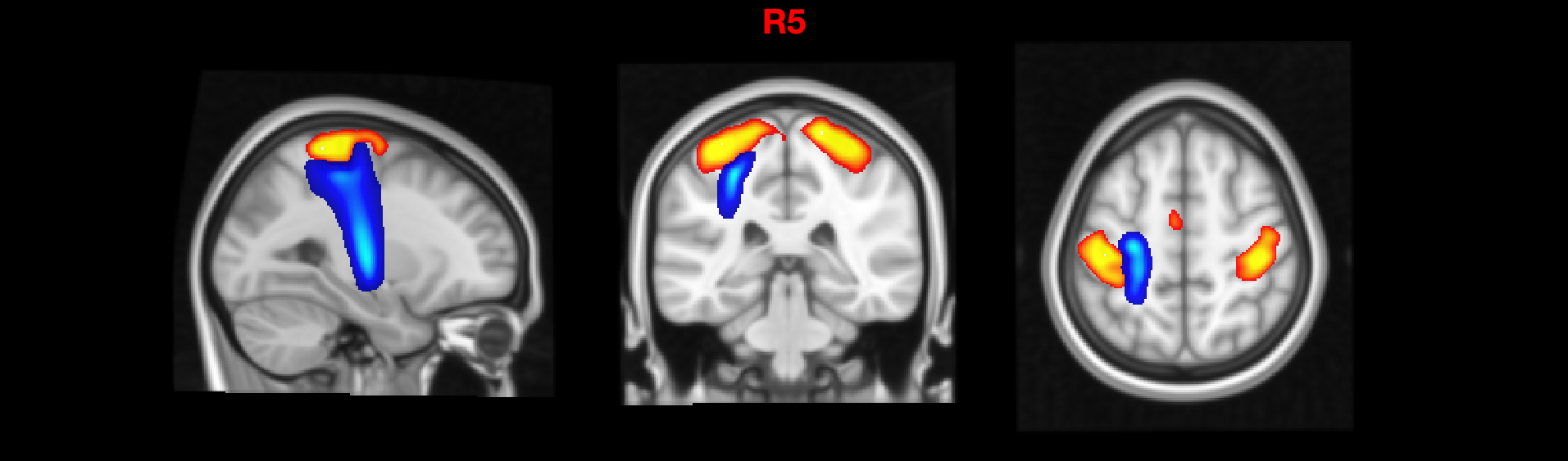

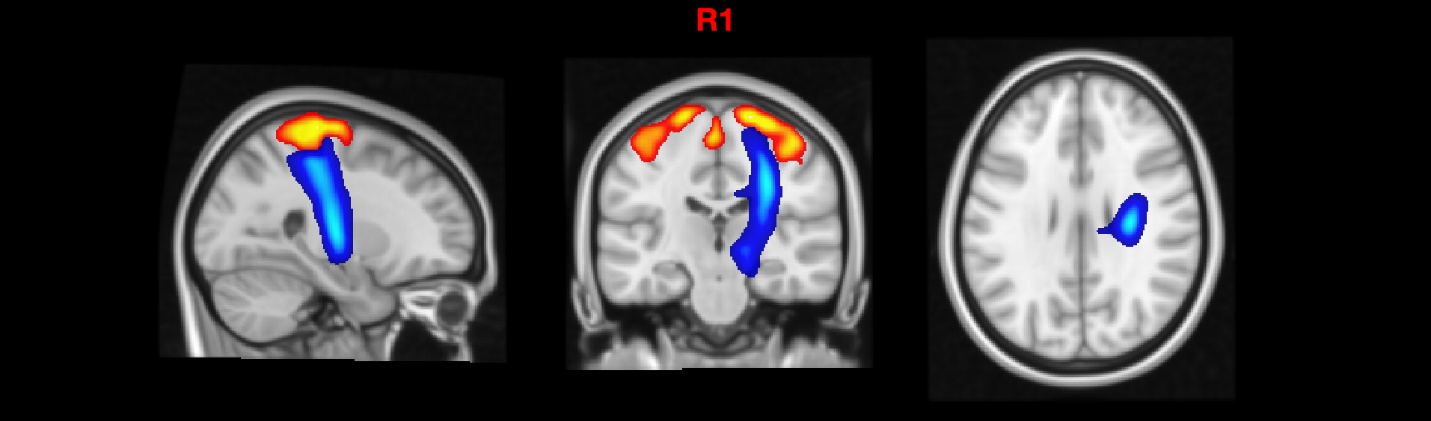

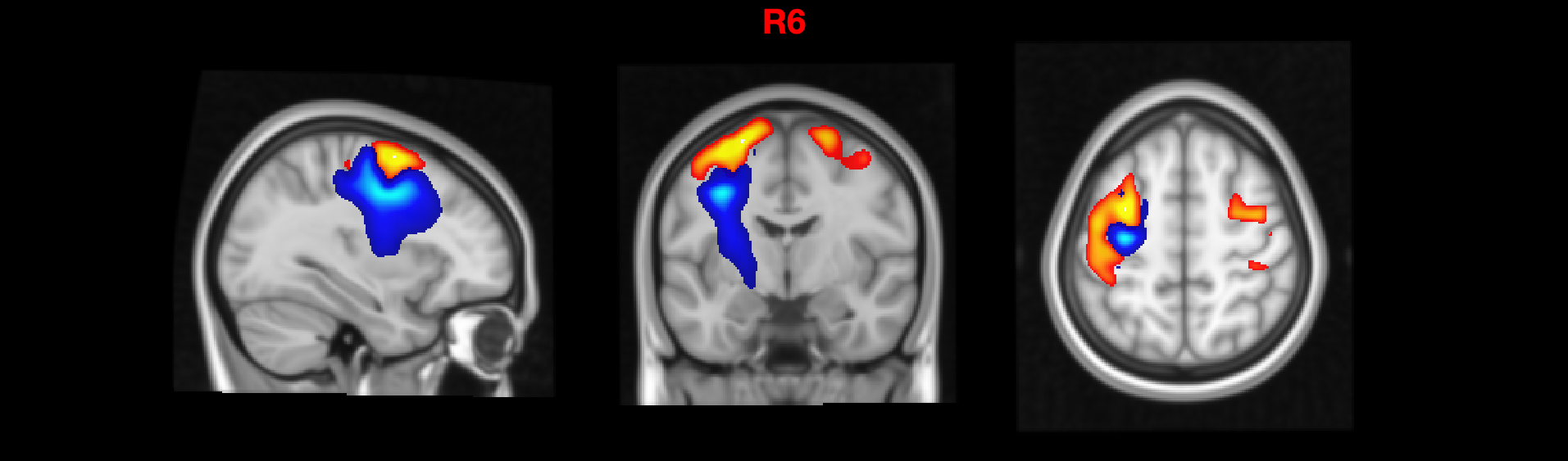

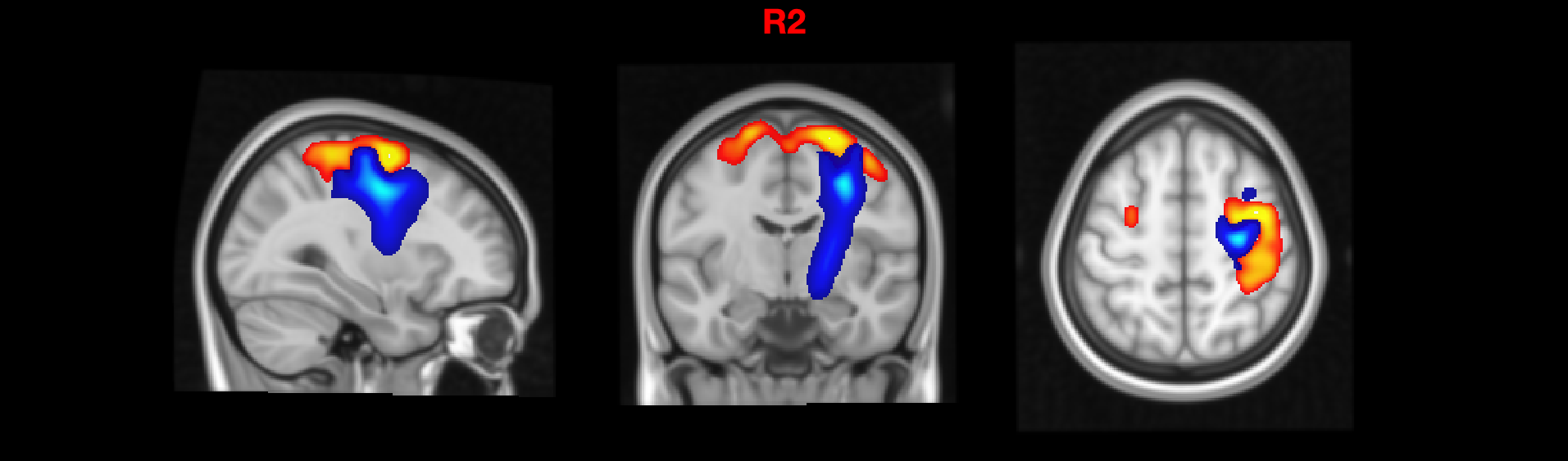


1. Source ­contribution and splitting


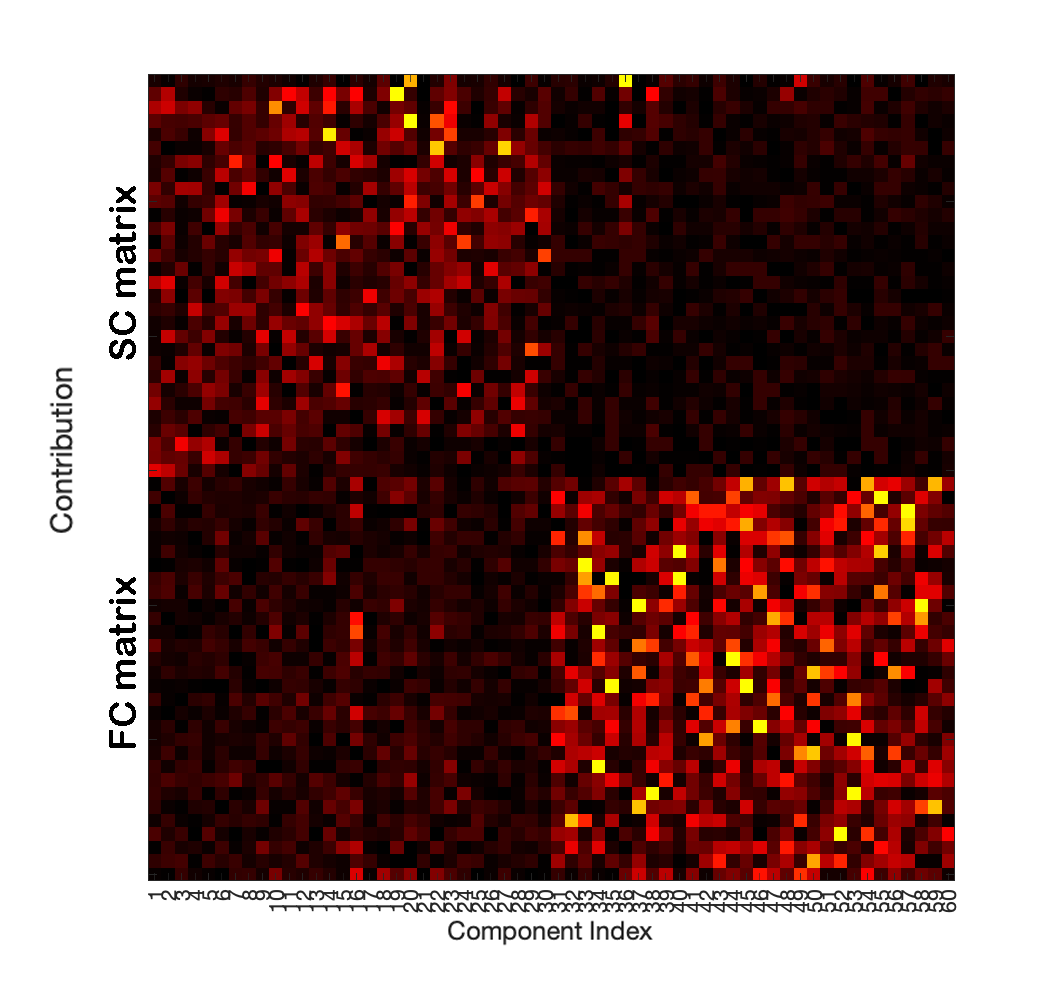

Supplement: Supplementary file 1 — APPENDIX S1 Supporting Information [file HBM-44-1533-s001.docx]
